# Flower Color Evolution and the Evidence of Pollinator-Mediated Selection

**DOI:** 10.3389/fpls.2021.617851

**Published:** 2021-07-26

**Authors:** Judith Trunschke, Klaus Lunau, Graham H. Pyke, Zong-Xin Ren, Hong Wang

**Affiliations:** ^1^CAS Key Laboratory for Plant Diversity and Biogeography of East Asia, Kunming Institute of Botany, Chinese Academy of Sciences, Kunming, China; ^2^Institute of Sensory Ecology, Heinrich-Heine-University, Düsseldorf, Germany; ^3^Department of Biological Sciences, Macquarie University, Ryde, NSW, Australia

**Keywords:** color perception, color preference, flower color variation, pollinator attraction, pollinator behavior, pollinator-mediated selection

## Abstract

The evolution of floral traits in animal-pollinated plants involves the interaction between flowers as signal senders and pollinators as signal receivers. Flower colors are very diverse, effect pollinator attraction and flower foraging behavior, and are hypothesized to be shaped through pollinator-mediated selection. However, most of our current understanding of flower color evolution arises from variation between discrete color morphs and completed color shifts accompanying pollinator shifts, while evidence for pollinator-mediated selection on continuous variation in flower colors within populations is still scarce. In this review, we summarize experiments quantifying selection on continuous flower color variation in natural plant populations in the context of pollinator interactions. We found that evidence for significant pollinator-mediated selection is surprisingly limited among existing studies. We propose several possible explanations related to the complexity in the interaction between the colors of flowers and the sensory and cognitive abilities of pollinators as well as pollinator behavioral responses, on the one hand, and the distribution of variation in color phenotypes and fitness, on the other hand. We emphasize currently persisting weaknesses in experimental procedures, and provide some suggestions for how to improve methodology. In conclusion, we encourage future research to bring together plant and animal scientists to jointly forward our understanding of the mechanisms and circumstances of pollinator-mediated selection on flower color.

## Introduction

There is an almost bewildering diversity of flower colors and color patterns in flowering plants with colors spanning the entire color spectrum of human and pollinator vision (Menzel and Shmida, 1993), and varying enormously over a range of geographic and temporal scales. Flower color shows, for example, differences at various spatial scales ranging from variation — both continuous and discrete — among individual plants of the same population, plant populations, closely related species, and different flowering communities (e.g., Menzel and Shmida, 1993; Meléndez-Ackerman et al., 1997; Irwin and Strauss, 2005; Ellis and Johnson, 2009; Caruso et al., 2010; Supplementary Table 1). Flower color may also show temporal variation within the same individual intrinsically as a result of aging or extrinsically in response to pollination or changes in abiotic conditions (e.g., Weiss, 1995; Suzuki and Ohashi, 2014; Supplementary Table 1).

There is wide agreement that today’s diversity of flower colors in angiosperms is largely shaped by variation in the interactions with pollinating animals through the process of natural selection (Schiestl and Johnson, 2013; Van der Niet et al., 2014; Gervasi and Schiestl, 2017). Cumulative evidence for this view arises from the following macro-evolutionary observations (see Supplementary Table 2 for relevant references): correlations between attributes of flowers and their flower visitors across lineages (i.e., pollination syndromes); pollinator shifts associated with transitions in flower color leading to geographic variation within or across plant lineages or entire plant communities; spatial variation in attraction of pollinators with different color preference resulting in local adaptation; effects of flower color on pollinator behavior causing disassortative mating and reproductive isolation in plant hybrid zones; and the resemblance of color signals of a floral or non-floral model by a mimicking plant (i.e., plant floral mimicry systems).

Pollinators can exert substantial selective pressure on flower color and drive the evolution of flower color signals through preferential visitation and pollination efficiency, because animal pollination involves an interaction between the various plant and floral attributes of flower color signaling on the one side, and the sensory abilities and behavioral responses of the potential pollinators on the other (Chittka and Menzel, 1992; Menzel and Shmida, 1993; Chittka and Raine, 2006). Variation in flower perception, detection and preferences by pollinators likely results in variable visitation, pollination success and male and/or female fitness among color phenotypes (e.g., Waser and Price, 1981; Campbell et al., 1997). Thus, the way pollinators perceive and discriminate differently colored flowers, and how they respond to the perceived differences through preferential visitation, may drive flower color divergence among morphs, populations or species (Campbell et al., 1997). Similarly, how pollinators perceive and respond to variation in flower color within populations should determine the target, shape and strength of pollinator-mediated selection (Waser and Price, 1981).

Demonstrating pollinators as agents of selection in natural populations is important because it can provide the missing linkage between variation in animal vision and color preferences, and macro-evolutionary patterns of variation in flower color in angiosperms. However, while much work has been done to document and explain variation in flower color among morphs within color di- or polymorphic populations, and among populations and species, quantification of continuous color variation within populations and possible selection on it is limited (Rausher, 2008; Sapir et al., 2021). Few studies have measured the form and strength of natural selection on flower color, and those that estimate the importance of pollinators for selection are rare (Table 1).

**TABLE 1:** Summary of estimates of selection on continuous flower color variation in published studies and the proportion of significant estimates shown in brackets.

Color Trait	*S*	β	γ_*ii*_	γ_*ij*_	Poll
Overall	6 (3)	18 (7)	10 (3)	6 (3)	12 (7)
Pigment concentration	1 (1)	4 (2)	3 (2)	1 (0)	3 (1)
Corolla color	2 (2)	6 (1)	2 (0)	2 (0)	2 (1)
Hue	1 (0)	5 (2)	3 (1)	2 (1)	4 (2)
Brightness	1 (0)	5 (2)	3 (1)	2 (0)	4 (2)
Saturation	1 (0)	4 (0)	2 (0)	1 (1)	3 (1)
Patterns/Nectar guides	2 (0)	4 (1)	3 (1)	1 (0)	3 (2)
UV pattern	0	2 (2)	1 (0)	1 (0)	2 (1)

*Poll corresponds to any study that determined pollinators as agent of selection through modeling of pollinator visitation or experimentation using a hand-pollination treatment. *S*, selection differential; β, directional selection gradient; γ_*ii*_, quadratic selection gradient; γ_*ij*_, correlational selection gradient.*

Pollinators can be expected to exert directional selection on flower color to increase detectability and stabilizing selection to increase pollinator constancy (Waser and Price, 1981; Chittka, 1997; Chittka et al., 1997). This is because, in natural populations, a flower color signal should serve two functions: First, it should contrast against the background for detectability by foraging pollinators (Giurfa et al., 1996; Menzel et al., 1997; Koski, 2020), and second, it should contrast against flowers of species co-occurring within the same community to ensure pollinator constancy and conspecific pollen transfer (Chittka and Menzel, 1992; Menzel and Shmida, 1993; Chittka, 1997; Chittka et al., 1997). Furthermore, because pollinators may choose flowers based on a set of phenotypic characters including, for example, inflorescence height and display size, flower color may also be subject to correlational selection favoring a combination of flower color with other pollinator attractive characters.

The form and strength of phenotypic selection in natural populations is typically quantified by regression analysis of relative fitness against standardized quantitative trait values across a large sample of individuals exhibiting substantial phenotypic variation (Price, 1970; Arnold and Wade, 1984). Here, the estimated equation takes the form of directional selection where the regression coefficient *S* (i.e., the selection differential in univariate analysis) or *β*_*i*_ (i.e., the selection gradient in multivariate models) is significantly < 0 indicating negative directional selection or significantly > 0 indicative for positive directional selection (Lande and Arnold, 1983). While selection differentials depict the total selection acting on a character, estimates of selection gradients control for possible covariances between correlated characters and therefore determine the direct targets of selection (Lande and Arnold, 1983). Non-linear selection is detected through second-order polynomial regression, whereby stabilizing selection occurs when the quadratic regression coefficient γ_*i*_ is significantly < 0, and disruptive selection when γ_*i*_ > 0 (Lande and Arnold, 1983; Stinchcombe et al., 2008). Furthermore, selection may favor phenotypic integration of two or more traits when selection gradients of trait combinations γ_*ij*_ are significantly different from 0 (Lande and Arnold, 1983; Phillips and Arnold, 1989).

In the context of pollination, the causes of selection (i.e., pollinators as selective agents) can be identified by either experimentation or modeling. For example, experimenting may be through manipulation of the pollination environment (i.e., a hand-pollination treatment to remove variation in fitness that is not associated with variation in the interaction with pollinators; Caruso et al., 2018; Sletvold, 2019). On the other hand, statistical estimation approaches may be used with structural equation modeling estimation (i.e., estimating the causal relationship of pollinator interactions with plant fitness; Souto-Vilarósa et al., 2018; Rodriguez-Castañeda et al., 2020; Brunet et al., 2021).

**FIGURE 1:**
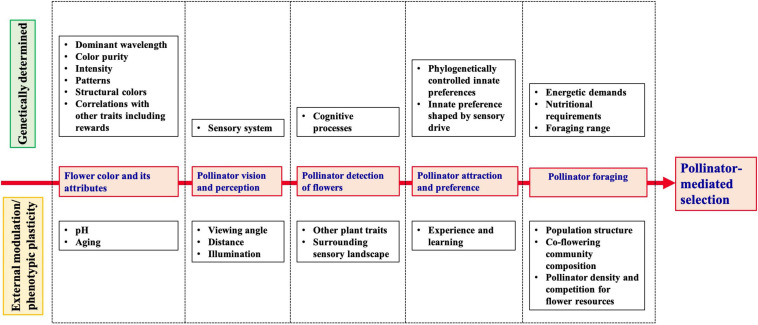
Schematic overview of the successive ways in the interaction of plants and pollinators involving flower colors, and how they impact on the outcome of pollinator-mediated selection on flower color.

In this review, we aim to summarize and discuss existing studies that have quantified selection on continuous flower color variation, and the evidence of pollinators as potential selective agents. In the first part, we synthesize the results of selection experiments in natural or experimental populations displaying continuous flower color variation. In the second part, we discuss the evidence for pollinator-mediated selection in light of characterization of flower color attributes and their variation, followed by pollinator visual and cognitive processing of the obtained signal (perception and detection of specific flower color attributes), the consequences for pollinator foraging behavior, and the context-dependence of selection (Figure 1). We highlight the gaps in knowledge and problems in experimental procedures, and provide suggestions for future directions. Here, we raise some of the unresolved questions to forward the understanding of how the interaction with pollinating animals may drive and maintain variation in flower color within and among natural populations.

## Review

In recent years, a number of studies have experimentally investigated the shape, strength and context-dependence of pollinator-mediated selection on flowering traits that mediate the interaction with pollinators in natural populations (reviewed in Caruso et al., 2018; Sletvold, 2019). Yet, we are aware of only 18 studies that aimed to quantify selection on continuous variation in petal coloration in the context of pollination (Tables 1, 2). Moreover, of these studies, only six used a hand-pollination treatment to estimate selection gradients associated with pollinator interactions (Caruso et al., 2010; Parachnowitsch and Kessler, 2010; Lavi and Sapir, 2015; Sletvold et al., 2016; Zhang et al., 2017; Souto-Vilarósa et al., 2018; Supplementary Table 3). Some other studies used modeling approaches linking the flower color phenotype - fitness relationship to pollinator visitation data to determine the contribution of pollinators to observed total selection (e.g., Veiga et al., 2015; Rodriguez-Castañeda et al., 2020; Brunet et al., 2021; Supplementary Table 3).

**TABLE 2:** Summary of linear phenotypic selection gradients (± SE) extracted from the literature for net selection (β_*C*_), non-pollinator-mediated selection (β_*HP*_) and pollinator-mediated selection (Δβ_*Poll*_) on flower color estimated in natural plant populations and experimental populations.

Species	Pollinator	Trait	*β*_*C*_	*β*_*HP*_	Δ*β*_*Poll*_	Reference
**Natural populations**						
*I.tenuituba, I. aggregata*	Hummingbirds, hawkmoths	Optical density	na	na	0.01 ± 0.12	Campbell et al. (1997)*
*Mimulus luteus*	Insects, hummingbirds	Guide shape CVA1	na	na	0.011 ± 0.06	Medel et al. (2003)*
*Mimulus luteus*	Insects, hummingbirds	Guide shape CVA2	na	na	−0.007 ± 0.06	
*Claytonia virginica*	Solitary bees	Corolla color	0.019	na	na	Frey (2004)
*W. albomarginata*	Solitary bees	R440/R530	na	na	0.179 ± 0.45	Campbell et al. (2012)*
*W.albomarginata*	Solitary bees	PC1 (Color hexagon distance)	na	na	−0.060 ± 0.44	
*Iris atropurpurea*, Year 1	Eucera bees	Anthocyanin conc.	0.203 ± 0.15	0.149 ± 0.17	0.054	Lavi and Sapir (2015)
*Iris atropurpurea*, Year 2	Eucera bees	Anthocyanin conc.	0.116 ± 0.09	**0.173 ± 0.09**	−0.057	
*Iris haynei*, Year 1	Eucera bees	Anthocyanin conc.	0.104 ± 0.13	0.081 ± 0.12	0.023	
*Iris haynei*, Year 2	Eucera bees	Anthocyanin conc.	0.087 ± 0.11	0.102 ± 0.11	−0.015	
*Gentiana lutea*	Bumblebees	PC1 (Yellowness)	**0.238 ± 0.08**	na	na	Veiga et al. (2015)
*Anacamptis morio*	Bumblebee queens	Brightness	0.28 ± 0.17	**−0.130 ± 0.04**	**0.42**	Sletvold et al. (2016)
*Anacamptis morio*	Bumblebee queens	Lip patch size	−0.25 ± 0.15	0.077 ± 0.04	−0.33	
*Anacamptis morio*	Bumblebee queens	Lip patch contrast	**0.50 ± 0.17**	−0.017 ± 0.04	**0.51**	
*Anacamptis morio*	Bumblebee queens	Lip spot area	−0.12 ± 0.14	0.018 ± 0.04	−0.14	
*Gymnadenia conopsea*	Butterflies, flies	PC7 (Corolla color)	−0.056 ± 0.12	na	na	Gross et al. (2016)
*Gymnadenia conopsea*	Butterflies, flies	PC7 (Corolla color)	−0.077 ± 0.08	na	na	
*Gymnadenia conopsea*	Butterflies, flies	PC7 (Corolla color)	−0.027 ± 0.06	na	na	
*Gymnadenia conopsea*	Butterflies, flies	PC7 (Corolla color)	−0.051 ± 0.05	na	na	
*Gymnadenia conopsea*	Butterflies, flies	PC7 (Corolla color)	0.021 ± 0.07	na	na	
*Gymnadenia conopsea*	Butterflies, flies	PC7 (Corolla color)	−0.085 ± 0.04	na	na	
*Gymnadenia conopsea*	Butterflies, flies	PC7 (Corolla color)	−0.090 ± 0.06	na	na	
*Caltha scaposa*	Bees, flies	UV bulls-eye size	−0.08 ± 0.06	**6.05 ± 0.88**	−6.13	Zhang et al. (2017)
*Caltha scaposa*	Bees, flies	UV proportion	0.14 ± 0.06	**−4.63 ± 0.68**	4.77	
*Iris lutescens*	Apoid bees	Anthocyanin conc.	**−0.19 ± 0.002**	n.s.	n.s.	Souto-Vilarósa et al. (2018)
*Iris lutescens*	Apoid bees	Flavonoid conc.	n.s.	n.s.	n.s.	
*Iris lutescens*, yellow	Apidae	Anthocyanin conc.	n.s.	n.s.	n.s.	
*Iris lutescens*, yellow	Apidae	Flavonoid conc.	**0.35 ± 0.01**	**na**	n.s.	
*Iris lutescens*, purple	Apidae	Anthocyanin conc.	n.s.	n.s.	n.s.	
*Iris lutescens*, purple	Apidae	Flavonoid conc.	**−0.20 ± 0.01**	**na**	n.s.	
*Iris pumila*, purple	Apidae	Anthocyanin conc.	n.s.	n.s.	n.s.	
*Iris pumila*, blue	Apidae	Anthocyanin conc.	**−0.25 ± 0.06**	**0.42± 0.003**	**−0.67**	
*Iris pumila*, yellow	Apidae	Anthocyanin conc.	na	na	na	
*Iris pumila*, purple	Apidae	Flavonoid conc.	n.s.	n.s.	n.s.	
*Iris pumila*, blue	Apidae	Flavonoid conc.	n.s.	n.s.	n.s.	
*Iris pumila*, yellow	Apidae	Flavonoid conc.	na	na	na	
*A.coriophora coriophora*	Bees	PC1 (RGB values)	0.002 ± 0.04	na	na	Joffard et al. (2020)
*A.coriophora coriophora*	Bees	PC1 (RGB values)	0.08 ± 0.12	na	na	
*A.coriophora coriophora*	Bees	PC1 (RGB values)	−0.02 ± 0.05	na	na	
*A.coriophora fragrans*	Bees	PC1 (RGB values)	−0.02 ± 0.06	na	na	
*A.coriophora fragrans*	Bees	PC1 (RGB values)	0.02 ± 0.05	na	na	
*A.coriophora martrinii*	Bees	PC1 (RGB values)	−0.04 ± 0.08	na	na	
*Silene littorea*	Bees, butterflies, *Hadena*	Anthocyanin conc.	−0.11 ± 0.10	na	na	Rodriguez-Castañeda et al. (2020)
*Silene littorea*	Bees, butterflies, *Hadena*	Corolla color	−0.1 ± 0.17	na	na	
*Medicago sativa*	Bees	Brightness	n.s.	na	na	Brunet et al. (2021)
*Medicago sativa*	Bees	Saturation	n.s.	na	na	
*Medicago sativa*	Bees	Hue	n.s.	na	na	
**Experimental populations**						
*I.tenuituba, I. aggregata*	Hummingbirds, hawkmoths	Optical density	na	na	**0.19 ± 0.05**	Campbell et al. (1997)*
*Lobelia siphilitica*	Bumblebees	Brightness	−0.050 ± 0.06	**0.111 ± 0.05**	**−0.161**	Caruso et al. (2010)
*Lobelia siphilitica*	Bumblebees	Saturation	−0.019 ± 0.06	0.074 ± 0.05	−0.093	
*Lobelia siphilitica*	Bumblebees	Hue	−0.019 ± 0.06	**0.101 ± 0.05**	−0.120	
*Penstemon digitalis*	Bumblebees	Nectar guide	−0.003 ± 0.01	−0.005 ± 0.01	0.002	Parachnowitsch and Kessler (2010)
*Penstemon digitalis*	Bumblebees	Nectar guide	−0.02± 0.03	na	na	Parachnowitsch et al. (2012)
*Lobelia siphilitica, H*	Bumblebees	Brightness	−0.062 ± 0.04	na	na	Wassink and Caruso (2013)
*Lobelia siphilitica, H*	Bumblebees	Saturation	−0.017 ± 0.04	na	na	
*Lobelia siphilitica, H*	Bumblebees	Hue	0.062 ± 0.04	na	na	
*Lobelia siphilitica, F*	Bumblebees	Brightness	−0.015 ± 0.03	na	na	
*Lobelia siphilitica, F*	Bumblebees	Saturation	0.115 ± 0.04	na	na	
*Lobelia siphilitica, F*	Bumblebees	Hue	−0.012 ± 0.03	na	na	
*Centaurea cyanus*	Bumblebees	PC1 (Brightness)	0.007	na	na	Renoult et al. (2013)
*Centaurea cyanus*	Bumblebees	PC2 (Blue-violet)	0.029	na	na	
*Centaurea cyanus*	Bumblebees	PC3 (UV)	**−0.061**	na	na	

Significant selection gradients at *P* < 0.05 are highlighted in bold. All selection gradients were estimated using multiple regression following the Lande and Arnold (1983) protocol. Multiple rows of the same species for the same trait correspond to different populations if not otherwise indicated. *Note that Campbell et al. (1997), Campbell et al. (2012), and Medel et al. (2003) estimated selection gradients based on pollinator visitations as fitness response rather than measurements of components of female fitness. *I*, *Ipomopsis*; *W*, *Wahlenbergi*a; *A*, *Anacamptis.*; F, Female; H, Hermaphrodite.

It is striking that studies have rarely detected significant evidence for directional net selection (which includes all possible causes of selection) on achromatic color parameters. In two studies, natural selection was found to act in a linear manner favoring less bright flowers in *Lobelia siphilitica* in one of these studies (Caruso et al., 2010), and brighter and contrast-rich petal colorization in the deceptive orchid *Anacamptis morio* in the other (Sletvold et al., 2016). In addition, the study by Sletvold et al. (2016) confirmed that pollinators accounted for 100% of observed net selection among open-pollinated plants. In addition, Brunet et al. (2021) found, in a population of *Medicago sativa*, that bumblebees (*Bombus impatiens*) preferred darker flowers but this was due to correlational selection with flower number. That bumblebees use achromatic flower color information (i.e., green contrast) and preferentially chose flowers with increased brightness resulting in significant directional selection, when modeled through the bees visual system, was also demonstrated by Renoult et al. (2013) in an experimental greenhouse setup, where bumblebees were freely foraging on potted cornflower, *Centaurea cyanus*.

Results of these studies contrast with the well-established finding from behavioral experiments that bees choose colors on the basis of chromatic signals and not their brightness (Ng et al., 2020). The above studies, that indicate the importance of color brightness for pollinator choice and foraging were all carried out in the wild or in large artificial populations. Laboratory studies, on the other hand, have indicated the importance of color purity (Lunau, 1990; Rohde et al., 2013). This inconsistency between laboratory behavioral tests and the behavior of wild bees awaits explanation (Ng et al., 2020).

There are few evidences for pollinators selecting on chromatic flower color traits (Table 2). In *Gentiana lutea*, which varies in flower color from yellow to orange, Veiga et al. (2015) found selection for increased yellowness as yellow flowers received higher pollinator visitation than flowers of alternative colors. In the study of *Medicago sativa* by Brunet et al. (2021) alfalfa leafcutting bees, *Megachile rotundata*, exerted stabilizing selection on hue, but neither *Apis mellifera* nor *Bombus impatiens* showed preferences for flower hue resulting in non-significant selection when summed over all bees.

Some studies have identified significant selection on achromatic or chromatic color signals, that was not clearly linked to the interactions with pollinators (e.g., Wassink and Caruso, 2013; Lavi and Sapir, 2015; Veiga et al., 2015; Souto-Vilarósa et al., 2018; Table 2). In *Gentiana lutea*, for example, flower yellowness significantly influenced both pollinator visitation and escape from seed predators, which resulted in significant net selection on flower color (Veiga et al., 2015). Wassink and Caruso (2013) detected significant net selection on saturation in *Lobelia siphilitica*, but only in hermaphrodite plants and only in the presence of co-flowering *Mimulus ringens*. In a population of *Iris pumilla*, Souto-Vilarósa et al. (2018) detected significant selection for increased anthocyanin concentration in the blue-flowered form, but no selection was detected in the purple morph. In an among-population study of selection in dimorphic *Iris lutescens*, Souto-Vilarósa et al. (2018) found some evidence for directional selection for increased flavonoid concentration in a yellow-flowered morph, and for decreased anthocyanin concentration consistent across the yellow- and purple-flowered morph. However, this was not a consistent pattern across other populations in which the two morphs co-occur.

Non-pollinator-mediated linear directional selection, as determined by experiment through a hand-pollination treatment, has been found in some studies (Caruso et al., 2010; Lavi and Sapir, 2015; Sletvold et al., 2016; Souto-Vilarósa et al., 2018; Table 2). Both Caruso et al. (2010) and Sletvold et al. (2016) detected selection on flower brightness among plants experimentally supplemented with pollen. Lavi and Sapir (2015) and Souto-Vilarósa et al. (2018) found selection, which was non-pollinator-mediated, for increased anthocyanin pigment concentration in *Iris atropurpurea* in one year of study and in *I. lutescens*, respectively. Further, Souto-Vilarósa et al. (2018) also found non-pollinator-mediated selection that was positive for flavonoid concentration in the yellow morph of *I. lutescens* and negative for the purple morph, suggesting disruptive selection within dimorphic populations.

Some studies have also found evidence for significant quadratic selection on flower color traits (Table 3). For example, in *Mimulus luteus* selection on nectar guide shape variables through pollinator visitation was disruptive (Medel et al., 2003), but it is unclear whether this translates into net selection through seed production. Both Lavi and Sapir (2015) and Souto-Vilarósa et al. (2018) found significant stabilizing selection on anthocyanin pigment concentration in *Iris atropurpurea* and the blue-flowered morph of *I. pumilla*, respectively, but selection was significantly associated with pollinator interactions only in the latter case.

**TABLE 3:** Summary of quadratic selection gradients (± SE) extracted from the literature for net selection (γ_*C*_), non-pollinator-mediated selection (γ_*HP*_) and pollinator-mediated selection (Δγ_*Poll*_) on flower color estimated in natural plant populations and experimental populations.

Species	Trait	γ_*C*_	γ_*HP*_	γ_*Poll*_	Reference
**Natural populations**					
*I. tenuituba, I. aggregata*	Optical density	na	na	0.03 ± 0.10	Campbell et al. (1997)*
*Mimulus luteus*	Guide shape CVA1	na	na	**6.338 ± 1.38**	Medel et al. (2003)*
*Mimulus luteus*	Guide shape CVA2	na	na	**2.580 ± 0.75**	
*Claytonia virginica*	Corolla color	−0.009	na	na	Frey (2004)
*W. albomarginata*	R440/R530	na	na	−0.091 ± 0.42	Campbell et al. (2012)*
*W. albomarginata*	PC1 (Color hexagon distance)	na	na	−0.563 ± 0.45	
*Iris atropurpurea*, Year 1	Anthocyanin conc.	−0.958 ± 1.99	0.018 ± 2.35	−0.976	Lavi and Sapir (2015)
*Iris atropurpurea*, Year 2	Anthocyanin conc.	1.121 ± 0.59	**1.329 ± 0.73**	−0.208	
*Iris haynei*, Year 1	Anthocyanin conc.	1.946 ± 2.43	2.266 ± 1.71	−0.320	
*Iris haynei*, Year 2	Anthocyanin conc.	−0.060 ± 1.04	−0.554 ± 0.99	0.494	
*Anacamptis morio*	Brightness	−0.270 ± 0.25	0.004 ± 0.06	−0.270	Sletvold et al. (2016)
*Anacamptis morio*	Lip patch size	0.320 ± 0.17	−0.022 ± 0.06	0.340	
*Anacamptis morio*	Lip patch contrast	−0.022 ± 0.26	−0.021 ± 0.06	−0.001	
*Anacamptis morio*	Lip spot area	0.074 ± 0.21	−0.002 ± 0.05	0.076	
*Iris lutescens, yellow*	Anthocyanin conc.	n.s.	n.s.	n.s.	Souto-Vilarósa et al. (2018)
*Iris lutescens, yellow*	Flavonoid conc.	n.s.	n.s.	n.s.	
*Iris lutescens, purple*	Anthocyanin conc.	n.s.	n.s.	n.s.	
*Iris lutescens, purple*	Flavonoid conc.	n.s.	n.s.	n.s.	
*Iris lutescens, yellow*	Anthocyanin conc.	n.s.	n.s.	n.s.	
*Iris lutescens, yellow*	Flavonoid conc.	n.s.	na	n.s.	
*Iris lutescens, purple*	Anthocyanin conc.	n.s.	n.s.	n.s.	
*Iris lutescens, purple*	Flavonoid conc.	n.s.	n.s.	n.s.	
*Iris pumila, purple*	Anthocyanin conc.	n.s.	n.s.	n.s.	
*Iris pumila, blue*	Anthocyanin conc.	n.s.	n.s.	n.s.	
*Iris pumila, purple*	Flavonoid conc.	n.s.	n.s.	n.s.	
*Iris pumila, blue*	Flavonoid conc.	−**0.900 ± 0.12**	0.003 ± 0.03	−**0.903**	
*Medicago sativa*	Brightness	n.s.	na	na	Brunet et al. (2021)
*Medicago sativa*	Saturation	n.s.	na	na	
*Medicago sativa*	Hue	n.s.	na	na	
**Experimental populations**					
*Penstemon digitalis*	Nectar guide	0.004 ± 0.01	**−0.006 ± 0.01**	0.01	Parachnowitsch and Kessler (2010)
*Centaurea cyanus*	PC1 (Brightness)	0.020	na	na	Renoult et al. (2013)
*Centaurea cyanus*	PC2 (Blue-violet)	0.030	na	na	
*Centaurea cyanus*	PC3 (UV)	0.003	na	na	

Significant selection gradients at *P* < 0.05 are highlighted in bold. All selection gradients were estimated using multiple regression following the Lande and Arnold (1983) protocol. *Note that Campbell et al. (1997), Campbell et al. (2012), and Medel et al. (2003) estimated selection gradients based on pollinator visitations as fitness response rather than measurements of components of female fitness. *I*, *Ipomopsis*; *W*, *Wahlenbergi*a; *A*, *Anacamptis.*

Evidence for correlational selection among flower color traits and other traits involved in pollinator interactions is generally scarce (Table 1). Few studies have estimated selection acting on combinations of different color attributes, or the integration of flower color with flower morphology, and no study has investigated a possible flower color - flower scent association as target of selection (Table 4). Among the four studies quantifying correlational selection (Medel et al., 2003; Campbell et al., 2012; Renoult et al., 2013; Brunet et al., 2021), three included flower size - flower color associations, two additionally included plant height - flower color associations, and one measured correlational selection on flower number and flower color. Brunet et al. (2021) detected significant selection for more flowers of higher saturation and reduced brightness by bumblebees in *Medicago sativa* but selection summed over all bees was only significant involving color saturation. And the study by Renoult et al. (2013) found selection for larger flowers of increased blue hue in *Centaurea cyanus*. This indicates that trait combinations involving flower color are important in at least some instances. However, most studies report weak correlations among flower color phenotype and flowering morphology, and consequently did not estimate correlational selection.

**TABLE 4:** Summary of correlational selection gradients (± SE) extracted from the literature for net selection (γ_*C*_), non-pollinator-mediated selection (γ_*HP*_) and pollinator-mediated selection (Δγ_*Poll*_) on flower color estimated in natural plant populations and experimental populations.

Species	Trait combination including flower color	γ_*C*_	γ_*HP*_	*γ_*Poll*_*	Reference
**Natural populations**					
*Wahlenbergia albomarginata*	R440/R530 × flower size	na	na	0.320 ± 0.35	Campbell et al. (2012)*
*Wahlenbergia albomarginata*	PC1 (Color hexagon distance) × flower size	na	na	0.013 ± 0.49	
*Mimulus luteus*	Guide shape CVA1 × plant height	na	na	0.058 ± 0.28	Medel et al. (2003)*
*Mimulus luteus*	Guide shape CVA1 × corolla size	na	na	−0.210 ± 0.30	
*Mimulus luteus*	Guide shape CVA1 × guide shape CVA2	na	na	0.187 ± 0.25	
*Mimulus luteus*	Guide shape CVA2 × plant height	na	na	0.226 ± 0.32	
*Mimulus luteus*	Guide shape CVA2 × corolla size	na	na	0.073 ± 0.30	
*Mimulus luteus*	Guide shape CVA2 × plant height	na	na	−0.564 ± 0.34	
*Mimulus luteus*	Guide shape CVA2 × corolla size	na	na	−0.332 ± 1.40	
*Medicago sativa*	Brightness × flowers per raceme	n.s.	na	na	Brunet et al. (2021)
*Medicago sativa*	Saturation × flowers per raceme	**0.370**	na	na	
*Medicago sativa*	Hue × flowers per raceme	n.s.	na	na	
*Centaurea cyanus*	PC1 (Brightness) × PC2 (Blue-violet)	0.004	na	na	Renoult et al. (2013)
*Centaurea cyanus*	PC1 (Brightness) × PC3 (UV)	−0.044	na	na	
*Centaurea cyanus*	PC1 (Brightness) × flower size	−0.021	na	na	
*Centaurea cyanus*	PC1 (Brightness) × plant height	−0.017	na	na	
*Centaurea cyanus*	PC2 (Blue-violet) × PC3 (UV)	−0.016	na	na	
*Centaurea cyanus*	PC2 (Blue-violet) × flower size	**0.101**	na	na	
*Centaurea cyanus*	PC2 (Blue-violet) × plant height	−0.038	na	na	
*Centaurea cyanus*	PC3 (UV) × flower size	−0.018	na	na	
*Centaurea cyanus*	PC3 (UV) × plant height	0.028	na	na	

Significant selection gradients at *P* < 0.05 are highlighted in bold. All selection gradients were estimated using multiple regression following the Lande and Arnold (1983) protocol. *Note that Campbell et al. (1997) and Medel et al. (2003) estimated selection gradients based on pollinator visitations as fitness response rather than measurements of components of female fitness.

In synthesis, it appears that pollinator-mediated selection on flower color is expected to influence its evolution, but is difficult to observe and quantify in natural populations. This difficulty arises, we suggest, because of several aspects in the interplay between plant color phenotypes and pollinator sensory ecology and foraging behavior. These include the complexity of flower color parameters to which pollinators can respond, mechanisms of animal color vision and behavioral flexibility as well as the environmental context (Figure 1).

Regarding pollinators, we suggest that the following factors may influence flexibility of pollinator preference expression for flower color when foraging in wild populations and weaken expected pollinator-mediated selection: (a) a prevalent unjustified assumption of innate fixed sensory capacities and preferences versus individual and learned acquisition; (b) poorly understood mechanisms and outcomes of flower color perception, discrimination and behavior and their interactions with the biotic and abiotic environment; and (c) an insufficient consideration of how pollinator visitation to plants and flowers is influenced by the likelihood of pollinator choices before any color preferences might come into play.

With respect to floral phenotypes, we similarly consider that the following diminish detectability of selection: (a) an insufficient quantification and discrimination of relevant aspects of flower color and color patterning; (b) a lack of considering functional relationships between flower color and other floral traits including the possible association with a floral resource such as nectar or pollen and their effects on pollinator foraging; (c) a poorly understood role of abiotic factors for determining variation in color among and within plant individuals.

In the following sections, we shall discuss these challenges arising in the context of animals foraging for floral resources involving flower color and attributes of the plants and flowers they visit.

### Flower Color Attributes, Its Variation and Function

**FIGURE 2:**
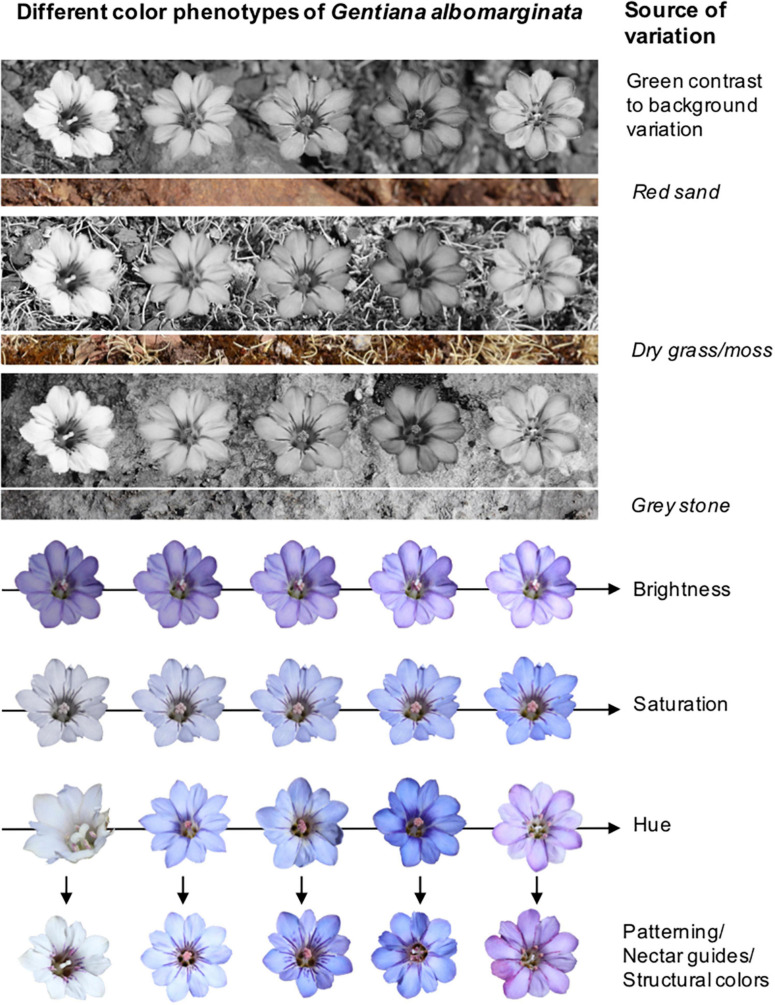
Representation of possible sources of variation in the color appearance of flowers exemplified in the alpine herb *Gentiana albomarginata* as a potential pollinator approaches flowers from far to near. Upper panels: Variation in green contrast between differently colored flowers and different backgrounds as a result of longwave photoreceptor perception. Lower panels: Variation in hue, saturation and brightness, which can be further modulated by variation in petal color patterning and nectar guides, or by variation in structural colors as here the presence and absence of fluorescent purple-colored pollen presented in male but not in female phase flowers.

The color appearance of a flower is determined by a complex interaction of chemical, physical and morphological factors on the one hand, and physiological and neural parameters on the other hand. A color phenotype as perceived by a flower visitor is predominantly characterized by its hue (the dominant spectral descriptor), saturation (the spectral purity) and brightness (the intensity of spectral reflectance), and also by the contrast with surrounding color (Bukovac et al., 2017; van der Kooi et al., 2019; Figure 2). Differences in colors among and within flowers are modulated through variation in the identity and concentration of plant pigments in distinct cell layers of the petals or other signaling organs (most commonly anthocyanins, flavonoids and carotenoids; van der Kooi et al., 2016), co-pigmentation (Yabuya et al., 2000; Mizuno et al., 2015) and epidermis cell shape (Kraaij and van der Kooi, 2019; Stavenga et al., 2020). In addition, structural colors (gloss, polarization, iridescence) and fluorescence can influence the appearance of flowers (Vignolini et al., 2013; van der Kooi et al., 2016). Iridescence, for example, can be considered to corrupt color identity, because the perceived color is dependent on the viewing angle (Kjernsmo et al., 2018). Similarly, fluorescence as often possessed by pollen (Mori et al., 2018) may also alter the perception of flower color, because pollen may thus absorb light in a distinct range of wavelength and reflect light in another range of wavelength, causing a bathochromic shift.

All such color components may have important signaling cues for pollinators (Lunau, 2000; Garcia et al., 2014; Horth et al., 2014; Verhoeven et al., 2018; van der Kooi et al., 2019). For example, colored pan traps used as artificial flower mimics often catch more flower-visitors if fluorescent colors are displayed (Shrestha et al., 2019), suggesting their potential role in pollinator attraction. The functional significance of structural colors as a signaling cue for pollinators has been shown in behavioral assays under laboratory conditions (Papiorek et al., 2014; van der Kooi et al., 2019). Yet, their importance for wild pollinators freely foraging in natural plant populations remains to be explored (Iriel and Lagorio, 2010; Garcia et al., 2019; Lunau et al., 2020). Further, trait manipulation experiments have demonstrated that masking of flower nectar guides significantly reduced pollinator visits (Hansen et al., 2012). However, details of color patterning as potential target of selection by pollinators have rarely been evaluated (but see Medel et al., 2003; Parachnowitsch and Kessler, 2010; Parachnowitsch et al., 2012; Sletvold et al., 2016; Zhang et al., 2017). That both Medel et al. (2003) and Sletvold et al. (2016) found significant selection on traits related to color patterning within flowers (Tables 2, 3) supports the view of their relevance for pollinator foraging decisions or pollination efficiency, and therefore may be more important than primary hue for pollinators selecting on flower color.

In previous studies, variation in flower colors have been measured in the following ways, each with advantages and disadvantages: by comparison of flower petals to color charts (three studies), digital photography (five), pigment extraction and spectrometry of extracts (three) or using direct spectral photometry of floral tissues in either laboratory or field (seven; Supplementary Table 3). With the exception of digital photography, a common limitation is that these methods offer little opportunity to separate and study details of flower color components such as the above described: patterning, transitions or contrasts within petals; nectar guides; anther and pollen colors; as well as aspects of polarization, florescence and shine. In addition, it may also be that some methods such as multiple color categories do not provide a sufficiently fine resolution to describe color variation in the analysis of selection. And even for commonly used spectrometry it may be difficult to reliably detect subtle differences in reflectance among individuals since even repeated measurements of the same flower can result in some deviances (Garcia et al., 2014; Johnsen, 2016).

It is important to separate different components of flower-visitor-subjective color appearance of flowers (e.g., hue, saturation, brightness, and other potentially visible parameters) for appropriately defining potential targets of selection, as evident from laboratory experiments. For example, bees presented with two yellow, less saturated colors and two blue, more saturated colors, preferentially chose the more saturated colors (i.e., higher spectral purity) regardless of the primary hue (yellow vs. blue; Papiorek et al., 2013; Rohde et al., 2013). Moreover, the few studies that have analyzed selection by separating hue, saturation and brightness, all suggest significant direct selection on one or multiple of such components (Tables 2, 3).

Various extrinsic factors can affect the appearance of colors and can thus influence pollinator visual perception of flowers, and consequently relationships between flower color and plant fitness. For example, many pollinators are active over a range of daylight and weather conditions (Corbet et al., 1993; Lawson and Rands, 2019), and may therefore experience temporal variation in illumination blurring intrinsic flower color identity based on pigmentation, which influences pollinator foraging (Arnold and Chittka, 2012). Furthermore, plants are exposed to spatial and temporal variation in microhabitats (e.g., direct sunlight or shade, darker or lighter environmental context), and so may display variation in color appearance or contrast with background independent of pigmentation (Arnold and Chittka, 2012). In addition, some plants exhibit intra-individual color changes (Weiss, 1995; Ohashi et al., 2015). To understand the impacts of the visual sensory context on pollinator-mediated selection requires to determine how such modulations in flower color perception may influence pollinator responses and the relationship of flower color and plant fitness.

**FIGURE 3:**
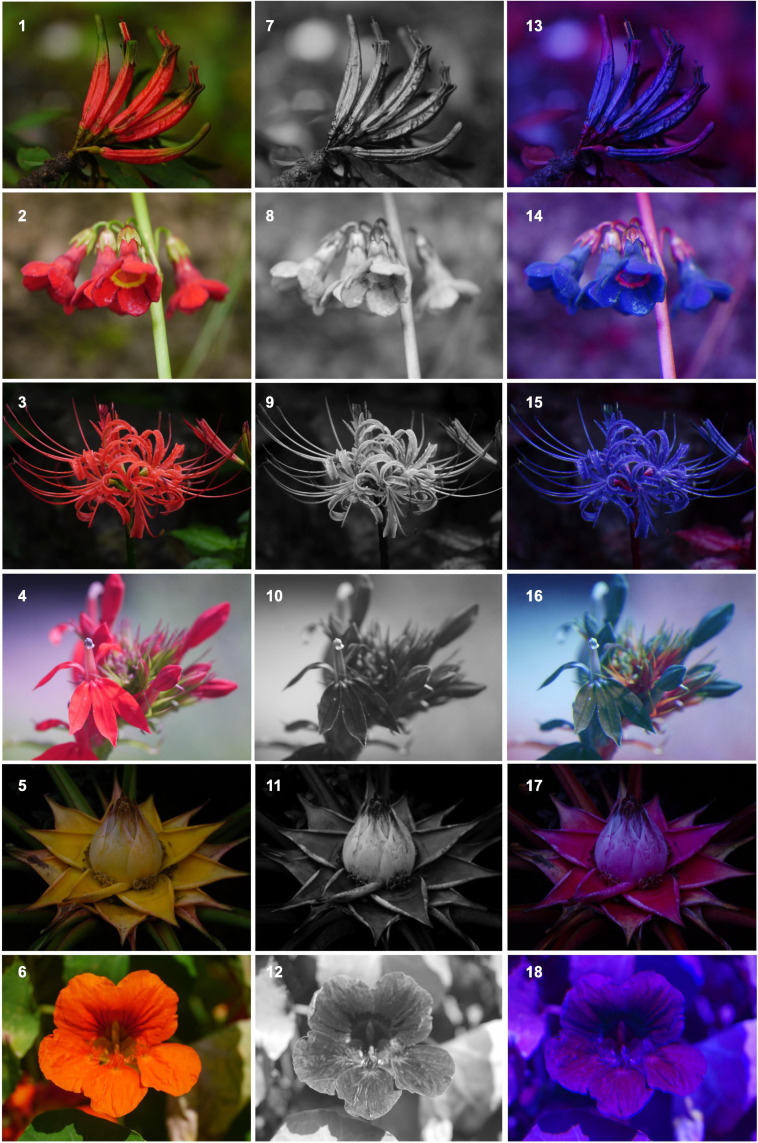
Illustration of flower colors associated with the bird pollination syndrome highlighting the weak visual contrast to the background in bee vision. Color photos show flowers seen by humans (1–7), in ultraviolet (8–12) whereas false color photos in bee view consider the bee-visible range of wavelength and display ultraviolet as blue, blue as green and green as red, and red is discarded, the mixed color ultraviolet-blue is displayed as blue-green, blue-green is displayed as yellow, and purple is displayed as blue (13–18; Verhoeven et al., 2018). See Figure 4 (2,8,14) and Figure 4 (4,10,16) for comparison of background color contrast in a bumblebee-pollinated plant. Illustrated species are by row from top to bottom: *Taxillus caloreas* (Loranthaceae), *Primula anisodora* (Primulaceae), *Lycoris radiata* (Amaryllidaceae), *Lobelia cardinalis* (Campanulaceae), *Musella lasiocarpa* (Musaceae), *Tropaeolum majus* (Tropaeolaceae).

**FIGURE 4:**
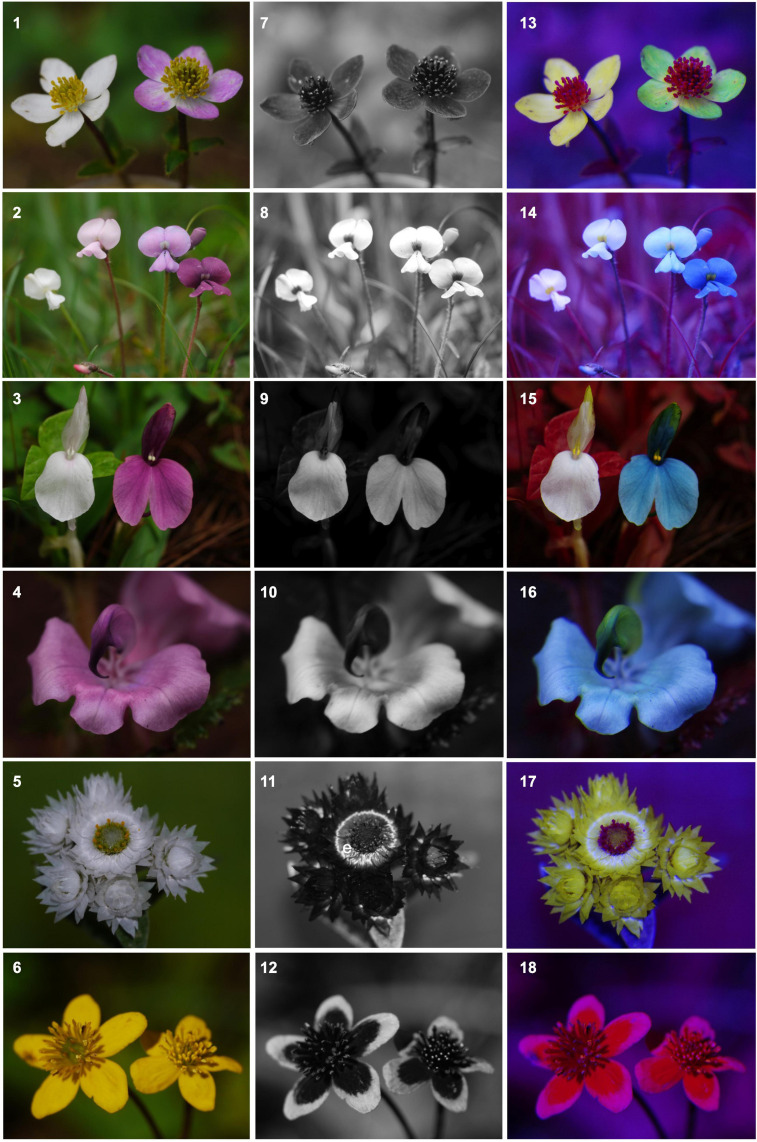
Variation of flower color seen through the eyes of humans (1–6), in ultraviolet (7–12) and seen through the eyes of bees (12–18) using false color photography illustrating potential causes of neglected variation in flower color and bias in choice of study system. For a description of color representation see legend of Figure 3. Illustrated species are by row from top to bottom: *Anemone trullifolia* var. *holophylla* (Ranunculaceae), *Tibetia yunnanensis* (Fabaceae), *Roscoea schneideriana* (Zingiberaceae), *Pedicularis superba* (Orobanchaceae), *Anaphalis nepalensis* (Asteraceae), *Caltha palustris* (Ranunculaceae).

Recent advances in digital photography and analytical software provide promising new avenues to characterize flower coloration in more detail (Garcia et al., 2014; Verhoeven et al., 2018). For example, Verhoeven et al. (2018), developed a false color photography technique, that combines digital image layers of flowers to visualize structural colors and access flower color variation as perceived by pollinators (Figures 3, 4). The use of this method recently allowed Lunau et al. (2020) to quantify shine across a large taxonomic sample of flowers, and the same procedure can also easily be applied to access within-population variation. Also, Hsu et al. (2018) used digital photography and sophisticated algorithms for faster digital image processing to quantify petal color gradients and spot patterns in *Sinningia speciosa.*

A further advantage of digital photography is the possibility to consider the color contrast between flower signal and the background. For example, integration of background color into the analysis of individual color variation has been successfully applied in areas of animal research such as habitat adaptation of body color in lizard (Stevens et al., 2007; Tong et al., 2019). This suggests that its application in the study of flower colors may also provide new insights for the understanding of selection and adaptation of flower color. Moreover, contrast to surrounding foliage or substrate has been shown to play an important role for pollinator visual perception and detection of flowers (Bukovac et al., 2017), but has so far not been considered in analysis of pollinator-mediated selection.

In some cases, there may also be concurrent differences in multiple attributes of coloration within flowers, making it difficult to determine whether pollinator responses are associated with variation in one factor or another. For example, many yellow-flowered angiosperms have an ultraviolet-absorbing center, sometimes referred to as ‘bull’s eye’ (Koski and Ashman, 2016). Alongside the UV-reflection, these bull’s eyes often include shifts in pigment concentration resulting in a possible covariation between color hue with changes in brightness, saturation and contrast against the surrounding outer petals (Koski and Ashman, 2013, 2014). In such cases the direct target of selection may not be easily identified, and experimental trait manipulations may be needed to disentangle the character underlying pollinator attraction and behavior (Campbell et al., 2014; Koski and Ashman, 2014).

In summary, we conclude that flower color is more complex than previously acknowledged in most selection studies, and suggest the following:

• A comprehensive characterization of flower colors requires the combined knowledge of chemistry and physics. Chemistry is necessary to understand pigment concentration and composition. Physics is required to understand absorption, transmission, light reflection and backscattering, and structural colors based on surface properties rather than on pigment layers (van der Kooi et al., 2016).
• Future research should be directed towards redefining pollinator behavior as responses to signals and targets of higher conspicuousness through spectral purity (i.e., higher color saturation rather than hue) and higher contrast against the background of the plant vegetative parts and the surrounding vegetation (specifically green contrast and color contrast).
• Within-flower color transitions and contrast seem also promising targets of pollinator-mediated selection, that should be increasingly explored.
• Finally, future studies may also test if structural color properties of flowers are direct targets of selection by animal pollinators and evolved to aid plant-pollinator communication.

### Pollinator Vision and Perception of Flower Color

The likelihood that a pollinator perceives a flower and eventually discriminates it from others will depend on complex interactions between various aspects of flower coloration, and the animals’ sensory system and associated cognitive abilities (Chittka and Menzel, 1992; Chittka and Raine, 2006). Pollinator detection of a flower will then depend on the distance and direction to the flower, on a variety of floral and plant attractive characters, and on the surrounding sensory landscape such as vegetation context, ambient weather and light conditions (Dyer and Chittka, 2004a; Chittka and Raine, 2006; Dyer, 2006; Skorupski et al., 2006).

**FIGURE 5:**
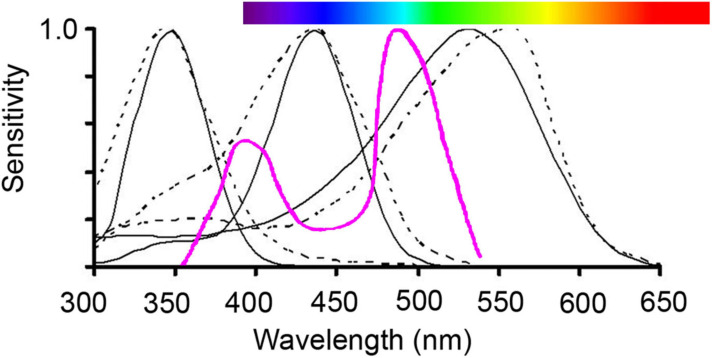
Illustration of trichromatic vision of important hymenopteran pollinators, bumblebees (solid line) and honeybees (dotted line), which have photoreceptor sensitivities peaking in the UV range (about 350 nm), in the blue range (about 440 nm) and in the green range (540 nm). Human color vision is given as a reference above in the horizontal spectral visible bar. The purple line (inverted Δλ/λ function for the honeybee; von Helversen, 1972; Chittka and Menzel, 1992) presents the two regions (400 nm and 500 nm), where spectral sensitivity curves overlap and therefore color discrimination can be expected to be highest. Graph reproduced from Shrestha et al. (2013).

How an animal perceives the color of a flower depends on the spectral sensitivity of its photoreceptors, the number of photoreceptors, and the neural processing of the received spectral signal (Dyer et al., 2011; van der Kooi et al., 2021). For example, it is now well established that Hymenopteran vision is shifted towards shorter wavelengths compared to human vision with peak sensitivities occurring at 340, 430, and 535 nm enabling most bee species to perceive UV (Peitsch et al., 1992, Supplementary Table 4; Figures 4, 5). The tetrachromatic vision in flies is based on two morphological receptor tandems (i.e., anatomically linked, consecutively laying pairs of photoreceptors), but they are lacking sensitivity to red light (Troje, 1993; An et al., 2018). Birds are generally tetrachromatic, but can be grouped into violet-sensitive species and UV-sensitive species, and both are sensitive to red light (Hart and Hunt, 2007). Butterflies’ vision is even more complex and diverse than this with trichromatic, tetrachromatic or even higher dimensional color vision occurring depending on the species (Arikawa, 2017). The spectral sensitivities of the photoreceptor types have been identified in some species belonging to each of the most common flower-visiting functional group including bees, butterflies, hawkmoths, flies, birds, bats, and beetles (see Supplementary Table 4 for an overview).

Thus, in contrast to other plant morphological characters, flower color is strictly speaking not simply a plants’ character, but a neural interpretation of the chemical and physical properties of a flower as interpreted through an animal’s visual system (i.e., a flower gets its colors only through the perceptual and cognitive process of its beholders; Garcia et al., 2020). For example, Figures 3, 4 illustrate flowers as seen by humans and by Hymenopterans with striking differences not only in hue but also in contrast among differently colored flower parts and contrast to green background foliage.

Models of animal vision can help us to understand how different kinds of pollinator functional groups might perceive flower color, and how similar or dissimilar two colors appear to a certain pollinator, and thus play a role for interpreting flower color discrimination and preferences. Models of animal vision have been formulated for a variety of pollinator taxa. For example, a pioneer achievement in this direction was the development of vision models to graphically represent the perceptual distances between loci in a color space, such as the frequently used color hexagon model for hymenopterans (Chittka, 1992; Chittka et al., 1994). The model visualizes the excitations from the three hymenopteran photoreceptors into a hexagon-shaped color space in the way that spectral reflectance data of flowers are transformed into units of discrimination in the trichromatic vision of a bee. Similar vision models have also been developed for flies (Troje, 1993) and butterflies (Koshitaka et al., 2008).

The vision models for hymenopteran, lepidopteran, and avian pollinators seem well aligned with results from behavioral studies (Vorobyev and Osorio, 1998; Vorobyev et al., 2001; Koshitaka et al., 2008; Kelber and Osorio, 2010), generally justifying their application in the study of pollinator-mediated selection. For example, four selection studies have used hexagon model transformation of spectral reflectance data or RGB values to estimate selection based on hexagon units of the color discrimination function. Moreover, Renoult et al. (2013) used this approach to show that selection on flower color brightness is a result of the hymenopteran visual system using a modeling approach (Supplementary Table S3).

However, models based on color spaces (e.g., color hexagon model) may be criticized as having low reliability, because they are based on a number of simplified and unrealistic assumptions and approximations (Osorio and Vorobyev, 2008; Shrestha et al., 2013). For example, although spectral sensitivity data are available for a diversity of bee species (Peitsch et al., 1992; Supplementary Table 4), most model calculations are done without using the specific spectral sensitivities of the investigated hymenopteran species, but by using the spectral sensitivity functions of the Western honeybee (*Apis mellifera*) or the buff-tailed bumblebee (*Bombus terrestris*) as an approximation. While some researchers argue that this generalized hexagon model of bee vision is likely applicable for a large number of hymenopteran pollinators (Briscoe and Chittka, 2001), because the spectral sensitivities of hymenopterans are (with few exceptions) similar (Peitsch et al., 1992; Supplementary Table 4), others argue it harbors severe limitations (Shrestha et al., 2013). Finally, it has been repeatedly demonstrated that the cognitive processes are not fixed but can be modulated in the course of foraging (Chittka et al., 2003; Dyer et al., 2012; further discussed below), and in response to spatially or temporally variable sensory landscapes (Koski, 2020). Therefore, color perception is perhaps more labile than any such vision model suggests.

Ideally, researchers would find that their results hold robust independent of the color vision model used (Telles and Rodríguez-Gironés, 2015; Gawryszewski, 2018), but so far such consistency has not always been found. For example, of those studies that have transformed flower color raw data into pollinator perception using the hexagon model (Campbell et al., 2012; Renoult et al., 2013; Joffard et al., 2020; Supplementary Table 3), some found no difference between selection estimated using vision model transformed data and raw data (Joffard et al., 2020), while others did find differences (Brunet et al., 2021). As far as we know, it is not known what explains these deviations (e.g., Brunet et al., 2021) and further research is needed to identify the factors governing such inconsistencies. Interestingly, Sletvold et al. (2016) and Brunet et al. (2021) found significant selection by pollinators on attributes of flower color without the use of any vision model but by demonstrating through supplemental hand-pollination and observation of pollinator behavior, respectively, that selection is a result of differential pollinator interactions.

Pollinators can only respond to variation among flower colors when they perceive the differences, and pollinator discrimination of certain colors may be constrained by the pollinator’s capacity for distinctive color perception. It has, for example, been demonstrated that bumblebees (*Bombus terrestris*) can learn to distinguish colors separated by 0.045 hexagon units (Dyer and Chittka, 2004b) and honeybees distinguish monochromatic stimuli separated by 4.5 nm (von Helversen, 1972). This suggests that Hymenopterans (the dominant pollinators in most selection studies) should be able to perceive the variation present in many natural plant populations of a single species (Campbell et al., 2012; Renoult et al., 2013).

However, these thresholds of minimal detectable differences were obtained under laboratory conditions and it is questionable whether such fine scale color discrimination applies to the foraging behavior of bees in natural floral communities with varying backgrounds and illuminations (Chittka and Thomson, 2001; Dyer and Chittka, 2004b; Dyer, 2006; Skorupski et al., 2006). For example, Dyer and Chittka (2004a) showed that illumination can affect bees color perception and the discrimination of fine color gradients: the number of correct choices of near similar colors increased with experience and with the color distance between the colors and was dependent on the ambient illumination as predicted by color vision model calculations.

Generally, laboratory behavioral experiments suggest, that fine color discrimination requires an association of the colored target with a reward. For example, depending on the experimental associations of color stimuli and rewards, Dyer and Chittka (2004b) showed that the ability of honeybees to discriminate similar colors can vary by about a factor of three. For free foraging bumblebees to specialize over flower colors, Chittka et al. (1997) suggest that, these colors must be separated by at least 0.1 hexagon units.

Yet, a strong consistent color-reward relationship as offered in laboratory settings is unlikely to occur in natural populations (Parachnowitsch et al., 2019; see discussion below). This may explain partly the deviation between the outcomes of experiments to identify the maximal possible visual capacities of bees, and those quantifying selection pressure on flower color in natural plant populations based on bee preferential flower visitation.

To help resolve the issues identified above in the context of quantification of pollinator-mediated selection, we make the following suggestions:

• Comparative spectral sensitivity data for model and non-model species of pollinators are needed. This would enable us to better understand the diversity of insect color perception, both among and within populations, before continuing to apply vision models. In worst case scenarios, such models only imprecisely fit the focal pollinator species.
• Measuring selection is combined with an approach to link variation in plant fitness to variation in pollinator preference and behavior (e.g., experimentally manipulating the pollination environment, structural equation modeling). It should then be possible to evaluate relationships between color phenotype and fitness (i.e., the target, mode and strength of selection), as mediated by pollinator interactions, without the use of vision models.

### Pollinator Attraction and Response to Flower Color

A pollinator may detect and respond to flower colors at a range of distances, with different color attributes coming into play as distances range from far to near, and ultimately when the pollinator reaches and moves onto a flower. For example, in bees, achromatic flower color is used for far distance detection of flowers, while chromatic flower color becomes important only when they are already in close proximity to them or flower targets are large-sized (Hempel de Ibarra et al., 2015). Only at relatively close distances (<10 cm), fine discrimination of chromatic colors between flowers is possible and enables expression of pollinator preferential choices to visit a specific flower phenotype (Giurfa et al., 1996; Hempel de Ibarra et al., 2015). Upon attraction, within-flower color patterns (e.g., floral guides) may become important through manipulating pollinator behavior close to or inside the flower (sensu Pohl et al., 2008; Pyke, 2016; Leonard and Papaj, 2011).

Different aspects of flower color may be subject to correlational selection through the effects on the number of fruits produced per plant and the seeds produced per flower. Flower achromatic and chromatic colors may determine the number of visits to a flower and thereby fruit production, while flower color patterning can influence pollinator orientation within the flower and flower handling time (Leonard and Papaj, 2011) as well as their feeding behavior such as the likelihood of proboscis extension (Hansen et al., 2012; Bischoff et al., 2015). Such patterns may act as ‘guides’ towards a potential resource (e.g., food, mating opportunity) and the reproductive organs, and thereby can influence the efficiency of pollen removal and deposition.

Pollinator responses to flower color signals may be innate, that is the spontaneous attraction of naïve animals as a consequence of their specific genetically determined visual systems (reviewed in Lunau and Maier, 1995). Such preferential innate attraction to certain color attributes is illustrated, by example, for preference of bees for blue or purple and some hoverflies for yellow signals (Lunau and Maier, 1995), while nocturnal moths’ preference for white flowers (Goyret et al., 2008) and hummingbirds’ preference for red flowers (Lunau et al., 2011) is not necessarily by innate response. Innate preferences are not necessarily associated with a specific hue. For example, a series of choice experiments with naïve bees and artificial flowers of varying colors have demonstrated that bees discriminate colors according to differences in hue, but spontaneously prefer colors of higher saturation independently of color hue (Lunau, 1990; Lunau and Maier, 1995; Lunau et al., 1996).

Several studies have demonstrated that almost any flower-visiting animal can be trained to respond to almost any flower color that is associated with a reward suggesting that most flower color preferences are extremely labile and can readily be changed through experience, and associative learning of a signal with a reward (Lunau and Maier, 1995; Goyret et al., 2008). For example, nectar-feeding hummingbirds learn to associate a particular flower color with relatively abundant nectar, and subsequently prefer to visit flowers with this color (Collias and Collias, 1968; Goldsmith and Goldsmith, 1979). A striking example is also provided by *Manduca* moths which innately prefer blue flowers but, just like other nocturnal pollinators, they are mainly seen foraging on white flowers in the wild (Goyret et al., 2008).

Labile color preferences have been shown for many animal pollinators including Hymenoptera, Lepidoptera, Diptera and Aves, allowing them to easily generalize in their foraging over a large array of flower colors (Lunau and Maier, 1995). This suggests that color preferences are dynamically formed and hence may exhibit variation that is temporal (Vaknin et al., 1996; Souza et al., 2018), or depends on flower patches of variable local co-flowering community (i.e., small scale spatial variation in preference; Wassink and Caruso, 2013).

In contrast to innate responses, pollinator color preference should depend on the flower color- reward relationship within the focal population and within the surrounding co-flowering community (Waser and Price, 1981; Campbell et al., 1997; Jersáková et al., 2016). If indeed flower color acts as a reliable signaling cue for pollinators in search for a reward, it provides a realistic mechanism explaining pollinator-mediated selection on flower color. However, none of the selection studies estimated the color-reward relationship and its temporal variation within the study population and the co-flowering community. Associations between color and nectar traits might be relevant across plant lineages explaining pollinator shifts, but may be less expected to occur within populations (Waser and Price, 1981; Parachnowitsch et al., 2019; Brunet et al., 2021). This is because pollinators are constantly depleting floral resources, flowers are refilling resources and there are temporal dynamics of flower abundance and community composition, and so it is unlikely that a consistent correlation between flower color and flower reward is prevalent in many plant populations and communities (Waser and Price, 1981). In fact, one of the arguments to explain the commonly found higher phenotypic variation in flowering traits among populations of non-rewarding species compared to populations of rewarding species is the lack of such a signal-reward association, which prevents pollinator learning of signals and consequently pollinator-mediated directional or stabilizing selection (Salzmann et al., 2007; Dormont et al., 2019). However, this hypothesis is contrasted by one of the few studies that detected significant pollinator-mediated selection on two flower color traits, which was conducted in an early-spring flowering non-rewarding orchid. This species begins to flower before any rewarding species comes to bloom and is pollinated by naïve bumblebee queens that have not yet acquired the ability to use a learned floral signal, yet there was significant selection by these bees for increased brightness and color contrast and the factors that maintain the large intra-population variation remain unknown (Sletvold et al., 2016).

The ability of animals to detect the color of plants and flowers depends on the visual context as well as the behavior of the animal. For example, Dyer and Chittka (2004a) showed that the ability to perceive and discriminate fine color differences (measured as the number of correct choices of near similar colors) decreased at lower illumination. However, this reduction in performance was less when bees were experienced and when the color distance between the stimuli was larger. Furthermore, the specific pollinators’ behavior can influence the detection when, for example, bees forfeit their abilities to discriminate colors in favor of making speedy decisions (Chittka et al., 2003; Skorupski et al., 2006).

We conclude that, the context-dependence of pollinator foraging challenges the linking of results of pollinator preferential behavior for flower colors as obtained under laboratory conditions to the interactions between natural plant and pollinator populations (Ng et al., 2020), and therefore suggest that:

• Future studies should test how results from laboratory studies are related to the foraging behavior of wild pollinators, and explore the factors that may explain possible deviations.
• Studies in the wild should further explore the context-dependence of pollinator preferential foraging using flower color in relation to experience and acquisition of color-reward relationships within flowering populations and communities.

### Pollinator-Mediated Selection on Flowering Traits

The direction, shape and strength of selection depends on, first, the functional relationships between traits and fitness, and second, the magnitude of variation in associated fitness. In the case of flower color, these relationships will be determined, as discussed above, by pollinator color perception, as well as their cognitive and behavioral responses. Therefore, the extent of within-population variation in flower color traits, and the way it affects variation in the interaction with pollinators, should determine the realized pollinator-mediated selection observed in natural populations. Consequently, the shape and strength of pollinator-mediated selection depends on the variation in the phenotypic character and its effect on pollen receipt and pollen export (Trunschke et al., 2020).

Pollinator-mediated selection will depend on the abilities of pollinators to discriminate between different colors and these may be influenced by a number of factors. For example, even if some bee pollinators do perceive differences in wavelength down to 0.045 hexagon units (see discussion in preceeding section; Dyer and Chittka, 2004b), this threshold of discrimination ability in bees is not equal over the spectral range of flower colors (Chittka and Waser, 1997; Figure 5). In general, pollinators should show highest discrimination capacities when two photoreceptors are maximum excited simultaneously, which explains the poor capacities of most bees to contrast red against their surroundings (Chittka and Waser, 1997; Figures 4, 5).

The spectral reflectance curves of flowers are striking because they possess sharp transitions in reflectance and because the position of these so-called ‘spectral reflectance’ marker points cluster in small ranges of wavelengths (Shrestha et al., 2013; Dorin et al., 2020; Figure 5). For example, it has been argued that hymenopteran spectral sensitivities of photoreceptor types are located in the wavelength spectrum such that they generate the largest possible range of different excitation values for inter-specific flower signals, and thus improve color discrimination among species within flowering communities (Chittka and Menzel, 1992). However, whether optimally foraging bees use fine-tuned color discrimination to distinguish between flower colors with similar marker points is not clear.

We may therefore expect that differential pollinator visitation is most expressed when intra-population color variation is either extremely large (e.g., Campbell et al., 1997; Hirota et al., 2013; Sletvold et al., 2016), or variation occurs in the range of maximum discrimination capacities. For example, selection in bee-pollinated plant species can be expected to be weak on chromatic signals in the yellowish to reddish range where subtle color variation cannot be well perceived (Chittka and Waser, 1997; Figure 5). This is in line with the lack of selection by bumblebee pollinators in several orange and yellow-flowered populations of *Gentiana lutea* (Sobral et al., 2015; Table 2). Further, if variation in flower color occurs in a range of the phenotypic distribution where it has little effect on the variation in fitness, this can result in weak selection limiting its detection (Trunschke et al., 2020).

Selection for flower colors could be stronger for plants with specialized pollinator interactions compared with plants utilized by pollinators belonging to several different taxonomic groups. It might be possible, for example, to predict the direction and shape of selection of flower color for specialized pollinator interactions, where there is a single main pollinator species with a particular operating visual system (Renoult et al., 2013). On the other hand, flower color selection may be less precise for plant species that are served by multiple pollinating animal species, which differ in their operating visual systems (Campbell et al., 1997), or their responses to perceived signals (Brunet et al., 2021).

Flower color may correlate with other traits influencing the number of pollinator visits and pollination efficiency (Gómez, 2000; Armbruster, 2002). Such traits may include overall plant stature and flower morphology (Gómez, 2000; Frey et al., 2011; Rodriguez-Castañeda et al., 2020), or, due to possible linkage in biosynthetic pathways, floral scent (Majetic et al., 2007; Zvi et al., 2008; Dormont et al., 2019 and references therein). Depending on the genetic variance-covariance matrix for all traits involved in pollinator interaction in a population, selection on flower color can therefore also act indirectly through pleiotropic links or in a correlative fashion favoring trait integration (Armbruster, 2002; Strauss and Whittall, 2006; Rausher, 2008).

Amongst a number of plant traits that may attract pollinators, one of the strongest predictors of pollinator visitation and pollination success is the size of floral displays (most commonly as the number of open flowers). This is because the number of flowers an individual plant produces strongly correlates with the quantity of flower rewards (Parachnowitsch et al., 2019), and therefore provides a reliable cue for pollinators to assess the amount of resource. Indeed, one study detected correlational selection between flower color and floral display by bumblebees favoring larger and darker flowers in *Medicago sativa* (Brunet et al., 2021). In contrast, most selection studies discussed here did not observe significant trait correlations between color and morphology, and have not explored this possibility further (Table 1). Few studies have compared selection differentials, which estimate both direct and indirect selection on a quantitative character, and selection gradients which reveal directional selection (Lande and Arnold, 1983; Arnold and Wade, 1984). These studies found that selection differentials are not largely different from selection gradients (Supplementary Table 5), suggesting that flower color is a direct target of selection in these studies.

In plants relying on animal pollination, the extent to which seed production is limited by pollination (i.e., the degree of pollen limitation) further influences the strength of pollinator-mediated selection. This is because pollen limitation influences the variation in fitness among individuals (i.e., the opportunity for selection; Sletvold and Ågren, 2014; Bartkowska and Johnston, 2015; Trunschke et al., 2017) and further, because pollen limitation can influence the trait-fitness relationship (discussed above). By definition pollen limitation quantifies the intensity of interactions with pollinators in the way that under high pollinator abundance, variation in pollination success among flowering individuals can be expected to be low, whereas under low pollinator abundancy the opposite occurs. Moreover, under high pollinator abundancy, competition among pollinators for floral resources may increase, which could lead pollinators to change their individual foraging strategy towards visiting an otherwise less preferred color phenotype (Waser and Price, 1981). Consistent with this prediction, the strongest selection on a flower color signal was found in the study population characterized by a high degree of pollen limitation (PL = 0.89 in the year of study; Sletvold et al., 2016). In other studies pollen limitation is often not reported, but fruit or seed set appears to be rather intermediate or high, which may explain weak detected selection.

In summary, we suggest that future studies should:

• Characterize functional relationships between flower color attributes and pollinator visitation and efficiency.
• Quantify how this translates into pollinator-mediated selection.
• Investigate the influence of the pollination environment including the rewarding co-flowering community and the magnitude of pollen limitation.

Such studies, with experimentally increased phenotypic variation, should elucidate the adaptive value of flower colors within contemporary populations, whereas manipulation of the environmental context should provide insights into the factors underlying variation in pollinators selecting for flower color.

## Conclusion

Quantifying selection acting on continuous flower color variation including the assessment of pollinator-mediated selection is necessary for understanding the importance of pollinator interactions for macro- and micro-evolutionary patterns and processes in the evolution and divergence of flower color in angiosperms. Clearly, more studies are needed that identify the direct targets, and characterize the direction, form and strength of selection on flower color signals within and among populations, and among species within flowering plant communities.

In this review, we have highlighted that while macro-evolutionary patterns for pollinator-driven evolution of flower color are well established and accepted, little is known about the underlying micro-evolutionary processes of pollinator-mediated selection within natural populations. Few studies have demonstrated that pollinators can be agents of selection on flower color, and detecting such selection appears to be challenging. This difficulty arises for multiple reasons related to the methodology of visualization and recognition of the color properties under selection, the likely flexibility of the trait-fitness relationship as response of pollinator perception, cognition and behavior, and the dependence of pollinator mediated selection on the pollination environment.

The evolution of floral signals in response to pollinator interactions is a complex field, that requires knowledge in botany, zoology, evolutionary biology, behavioral ecology as well as sensory physiology and neurobiology. Similar to floral scent blends, flower color should be recognized as a receiver-dependent and complex trait, the study of which requires an interdisciplinary approach. To date, this is seldomly played out in practice, and we strongly encourage scientists from animal and plant research to cross borders and work collaboratively.

## Author Contributions

All authors participated in the initial discussion for the manuscript. JT drafted a first version and led the writing, and all authors revised subsequent versions.

## Conflict of Interest

The authors declare that the research was conducted in the absence of any commercial or financial relationships that could be construed as a potential conflict of interest.

## Publisher’s Note

All claims expressed in this article are solely those of the authors and do not necessarily represent those of their affiliated organizations, or those of the publisher, the editors and the reviewers. Any product that may be evaluated in this article, or claim that may be made by its manufacturer, is not guaranteed or endorsed by the publisher.
