# Supplementary material for: Flower Color Evolution and the Evidence of Pollinator-Mediated Selection
Source: Front Plant Sci. 2021 Jul 26;12:617851. doi: 10.3389/fpls.2021.617851 (PMC8350172; doi:10.3389/fpls.2021.617851)
Supplement: Supplementary file 1 [file Data_Sheet_1.docx]

**Table S1:** Literature examples describing variation in flower color at various levels ranging from community-wide variation to intra-individual variation.

| **Level of** | **Plant study system** | **Plant family** | **Reference** |
| --- | --- | --- | --- |
| **Variation** |  |  |  |
|  |  |  |  |
| ***Community-wide*** | |  |  |
|  | 198 species |  | Menzel and Shmida (1993) |
|  | 168 species |  | Gumbert et al. (1999) |
|  | 74 species |  | Arnold et al. (2009) |
|  | 17 species |  | Newman et al. (2014) |
|  | 244 species | 76 families | Makino and Yokoyama (2015) |
|  | 39 species | 14 families | Van der Kooi et al. (2016) |
|  | 135 species | 27 families | Binkenstein et al. (2017) |
|  | 41 species | 14 families | Kantsa et al. (2017) |
|  | 71 species | 27 families | Bergamo et al. (2018) |
|  |  |  |  |
| ***Among sister species*** | |  |  |
|  | *Ipomopsis aggregata, I. tenuituba* | Polemoniaceae | Méléndez-Ackerman et al. (1997) |
|  | *Mimulus lewisii, M. cardinalis* | Phrymaceae | Schemske and Bradshaw (1999) |
|  | 24 *Oxalis* communities | Oxalidaceae | De Jager et al. (2010) |
|  | *Anserina pacifica, A. anserina* | Rosaceae | Koski and Ashman (2013) |
|  | 177 species of Potentilleae | Rosaceae | Koski and Ashman (2016) |
|  | 3 *Anacamptis coriophora* ssp. | Orchidaceae | Joffard et al. (2020) |
|  | 76 daisy species | Asteraceae | Kemp et al. (2019) |
|  |  |  |  |
| ***Among populations*** | |  |  |
|  | *Gorteria diffusa* | Asteraceae | Ellis and Johnson (2009) |
|  | *Anserina pacifica, A. anserina* | Rosaceae | Koski and Ashman (2013) |
|  | *Gentiana lutea* | Gentianaceae | Sobral et al. (2015), Veiga et al. (2015) |
|  | *Traunsteineria globosa* | Orchidaceae | Jersákova et al. (2016) |
|  |  |  |  |
| ***Within populations*** | |  |  |
| ***discrete*** |  |  |  |
|  | *Raphanus sativus* | Brassicaceae | Irwin and Strauss (2005) |
|  | *Gentiana lutea* | Gentianaceae | Sobral et al. (2015) |
|  | *Iris pubescens, I. pumilla* | Iridaceae | Souto-Vilarósa et al. (2017) |
|  | 148 orchid species | Orchidaceae | Dormont et al. (2019) |
|  |  |  |  |
| ***continuous*** |  |  |  |
|  | *Lobelia siphilitica* | Campanulaceae | Caruso et al. (2010) |
|  | *Wahlenbergia albomarginata* | Campanulaceae | Campbell et al. (2012) |
|  | *Anserina pacifica, A. anserina* | Rosaceae | Koski and Ashman (2013) |
|  | *Iris atropurpurea* | Iridaceae | Lavi and Sapir (2015) |
|  | *Iris haynei* | Iridaceae | Lavi and Sapir (2015) |
|  | *Anacamptis morio* | Orchidaceae | Sletvold et al. (2016) |
|  | *Anacamptis coriophora* subspecies | Orchidaceae | Joffard et al. (2020) |
|  | *Clarkia unguiculata* | Onagraceae | Peach et al. (2020) |
|  |  |  |  |
| ***Within-individual*** | |  |  |
|  | 393 species | 77 families | Weiss (1995) |
|  | *Weigela coraeensis* | Caprifoliaceae | Suzuki and Ohashi (2014) |
|  | Survey across 219 species | 14 families | Ohashi et al. (2015) |
|  | *Arnebia szechenyi* | Boraginaceae | Zhang et al. (2017) |

**Table S2:** Literature examples documenting the role of pollinators in driving macro-evolutionary patterns and processes of flower color in angiosperms.

| **Level** | ***Study System*** | **Pollinators** | **Reference** |
| --- | --- | --- | --- |
|  |  |  |  |
| ***Pollination syndromes involving flower color*** | |  |  |
|  |  |  | Faegri and van der Pjil (1971) |
|  |  |  | Fenster et al. (2004) |
|  |  |  |  |
| ***Pollinators shifts asscociate with a shift in flower color*** | |  |  |
|  | *Mimulus lewisii, M. cardinalis* | Hummingbirds, bees | Schemske and Bradshaw (1999) |
|  | *Mimulus aurantiacus clades* | Hummingbirds, hawkmoth | Streisfeld and Kohn (2007) |
|  | *Oxalis communities* | Honeybees | De Jager et al. (2011) |
|  | *Hemerocallis fulva, H. citrina* | Butterfly, hawkmoth | Hirota at al. (2012) |
|  | *17 species pollinator guild* | Prosoeca lingipennis | Newman et al. (2014) |
|  | *Daisy communities* | Flies | Kemp et al. (2019) |
|  |  |  |  |
| ***Local adaptation to pollinators involving flower color*** | |  |  |
|  | *Mimulus lewisii, M. cardinalis* | Hummingbirds, bees | Schemske and Bradshaw (1999) |
|  | *Mimulus aurantiacus clades* | Hummingbirds, hawkmoth | Streisfeld and Kohn (2007) |
|  | *Disa ferruginea ecotypes* | Butterflies | Newman et al. (2012) |
|  |  |  | Newman et al. (2014) |
|  |  |  |  |
| ***Dissasortative mating and reproductive isolation based on flower color*** | | |  |
|  | *Ipomopsis aggregata, I. tenuituba* | Hummingbirds, hawkmoth | Campbell et al. (1997) |
|  | *Ipomopsis aggregata, I. tenuituba* | Hummingbirds, hawkmoth | Aldridge and Cambell (2007) |
|  | *Antirrhinum majus subspecies* | Bees | Tastard et al. (2008) |
|  | *Phlox drummondii, P. cuspidata* |  | Hopkins and Rausher (2012) |
|  | *Mimulus aurantiacus clades* | Hummingbirds | Handelman and Kohn 2012() |
|  |  |  |  |
| ***Flower mimikry involving flower color*** | |  |  |
|  | *Gorteria diffusa* | Bombylid fly | Johnson and Midgley (1997) |
|  | *Disa ferruginea* | Butterflies | Newman et al. (2012) |
|  | *Disa communities* | Prosoeca ganglbaueri | Whithead et al. (2018) |

**Table S3:** Summary of methodological approaches taken in published studies to quantify flower color variation, pollinator color perception and the selective agent. HM = Hexagon model for bee vision (Chittka, 1992); HPT = Hand-pollination treatment (Sandring and Ågren, 2009); SEM = Structural equation modelling (Grace, 2006); DSD = Distribution selection gradient modelling (Henshaw and Zemel, 2017); AM = Aster modelling (Geyer et al., 2007; Shaw et al., 2008); * Note that Campbell et al., 1997, Medel et al. (2003) and Campbell et al., 2012 estimate selection with flower visitation as fitness response variable.

|  | **Method approach** | | |  |
| --- | --- | --- | --- | --- |
| **Species** | **Color quantification** | **Vision model** | **Agent of selection** | **Reference** |
| *Ipomopsis tenuituba, I. aggregata* | Petal pigment extracts | no | yes* | Campbell et al. (1997) |
| *Mimulus luteus* | Digital photography | no | Yes* | Medel et al. (2003) |
| *Claytonia virginica* | Spectral reflectance | no | no | Frey (2004) |
| *Lobelia siphilitica* | Spectral reflectance | no | HPT | Caruso et al. (2010) |
| *Penstemon digitalis* | Colour scale 4 levels | no | HPT | Parachnowitch and Kessler (2010) |
| *Wahlenbergia albomarginata* | Spectral reflectance | HM | yes* | Campbell et al. (2012) |
| *Penstemon digitalis* | Colour scale 4 levels | no | no | Parachnowitch et al. (2012) |
| *Lobelia siphilitica* | Spectral reflectance | no | no | Wassink and Caruso (2013) |
| *Iris atropurpurea, I. haynei* | Petal pigment extract | no | HPT | Lavi and Sapir (2015) |
| *Gentiana lutea* | Spectral reflectance | no | SEM | Veiga et al. (2015) |
| *Centaurea cyanus* | Spectral reflectance | HM | no | Renoult et al. (2015) |
| *Anacamptis morio* | Digital photography | no | HPT | Sletvold et al. (2016) |
| *Gymnadenia conopsea* | Colour scale 6 levels | no | no | Gross et al. (2016) |
| *Caltha scaposa* | Digital photography | no | HPT | Zhang et al. (2017) |
| *Iris lutescens, I. pumilla* | Petal pigment extract | no | HPT, AM | Souto-Vilarosa et al. (2018) |
| *Anacamptis coriophora* spp. | Digital photography | HM and none | no | Joffard et al. (2020) |
| *Silene littorea* | Digital photography | no | SEM | Rodriguez-Castañeda et al. (2020) |
| *Medicago sativa* | Spectral reflectance | HM and none | DSD | Brunet et al. (2021) |

**Table S4:** Examples of spectral sensitivities representing the diversity of flower visiting animals.

| **Species** | **Family** | **Visual system** | **ERG spectral sensitivity** | **Reference** |
| --- | --- | --- | --- | --- |
|  |  |  |  |  |
| **Coleoptera** |  |  |  |  |
| *Cetonia aurata* | Scarabaeidae, Scarabaeoidae | dichromatic | 350 (UV), 530 (green) | Frantsevich (1977) |
| *Pygopleurus israelitus* | Glaphyridae, Scarabaeoidea | trichromatic | 360 (UV), 517 (green), 631 (red) | Martinez-Harms et al. (2012) |
|  |  |  |  |  |
| **Diptera** |  |  |  |  |
| *Eristalis tenax* | Syrphidae | dichromatic | 350 (UV), 450 (blue-green) | Horridge et al. (1975) |
| *Glossina morsitans* | Glossinidae | dichromatic | 360 (UV), 500 (green) | Hardie et al. (1986) |
|  |  |  |  |  |
| **Hymenoptera** |  |  |  |  |
| *Apis mellifera* | Apidae | trichromatic | 345 (UV), 440 (blue), 550 (green) | Von Helversen (1972) |
| *Melipona quadrifasciata* | Apidae | trichromatic | 340 (UV), 450 (blue), 540 + 360 (green) | Hertel and Ventura (1985) |
| *Melipona marginata* | Apidae | trichromatic | 340 (UV), 450 (blue), 540 + 360 (green) | Hertel and Ventura (1985) |
| *Trigona spinipes* | Apidae | trichromatic | 340 (UV), 540 (green) | Hertel and Ventura (1985) |
| *Osmia rufa* | Megachilidae | trichromatic | 360 (UV), 430 (blue), 580 (green) | Menzel et al. (1986) |
| *Anthidium manicatum* | Apioidae, Apidae | trichromatic | 324 (UV), 440 (blue), 532 (green) | Peitsch et al. (1992) |
| *Bombus hypnorum* | Apioidae, Apidae |  | 524 (green) | Peitsch et al. (1992) |
| *Bombus mori* | Apioidae, Apidae | trichromatic | 352 (UV), 440 (blue), 548 (green) | Peitsch et al. (1992) |
| *Bombus monticula* | Apioidae, Apidae | trichromatic | 336 (UV), 440 (blue), 544 (green) | Peitsch et al. (1992) |
| *Bombus jonellus* | Apioidae, Apidae | trichromatic | 336 (UV), 432 (blue), 544 (green) | Peitsch et al. (1992) |
| *Bombus terrestris* | Apioidae, Apidae | trichromatic | 328 (UV), 428 (blue), 536 (green) | Peitsch et al. (1992) |
| *Bombus lapidarius* | Apioidae, Apidae | trichromatic | 332 (UV), 432 (blue), 544 (green) | Peitsch et al. (1992) |
| *Apis mellifera* | Apioidae, Apidae | trichromatic | 344 (UV), 436 (blue), 544 (green) | Peitsch et al. (1992) |
| *Lestrimelitta limao* | Apioidae, Meliponidae |  | 536 (green) | Peitsch et al. (1992) |
| *Trigona spinipes* | Apioidae, Meliponidae | trichromatic | 340 (UV), 440 (blue), 536 (green) | Peitsch et al. (1992) |
| *Melipona marginata* | Apioidae, Meliponidae | trichromatic | 340 (UV), 450 (blue), 540 (green) | Peitsch et al. (1992) |
| *Melipona quadrifasciata* | Apioidae, Meliponidae | trichromatic | 356 (UV), 428 (blue), 528 (green) | Peitsch et al. (1992) |
| *Schwarziana sp.* | Apioidae, Anthophoridae | trichromatic | 343 (UV), 440 (blue), 528 (green) | Peitsch et al. (1992) |
| *Nomada alboguttata* | Apioidae, Anthophoridae |  | 428 (green), 512 (green) | Peitsch et al. (1992) |
| *Proxylocopa sp.* | Apioidae, Anthophoridae | trichromatic | 312 (UV), 424 (blue), 532 (green) | Peitsch et al. (1992) |
| *Xylocopa brasilianum* | Apioidae, Anthophoridae | trichromatic | 360 (UV), 428 (blue), 544 (green) | Peitsch et al. (1992) |
| *Melecta punctata* | Apioidae, Anthophoridae | trichromatic | 336 (UV), 428 (blue), 540 (green) | Peitsch et al. (1992) |
| *Anthophora acervorum* | Apioidae, Anthophoridae | trichromatic | 348 (UV), 428 (blue), 528 (green) | Peitsch et al. (1992) |
| *Chelostoma florisomme* | Apioidae, Megachilidae |  | 324 (UV), 548 (green) | Peitsch et al. (1992) |
| *Osmia rufa* | Apioidae, Megachilidae | trichromatic | 344 (UV), 432 (blue), 560 (green) | Peitsch et al. (1992) |
| *Callonychium petuniae* | Apioidae, Andrenidae | tetrachromatic | 346 (UV), 430 (blue), 535 (green), 600 (red) | Peitsch et al. (1992) |
| *Andrena florea* | Apioidae, Andrenidae | trichromatic | 340 (UV), 412 (blue), 536 (green) | Peitsch et al. (1992) |
| *Oxea flavescens* | Apioidae, Oxeidae | trichromatic | 370 (UV), 435 (blue), 536 (green) | Peitsch et al. (1992) |
| *Lasioglossum albipes* | Apioidae, Halictidae |  | 516 (green) | Peitsch et al. (1992) |
| *Lasioglossum malachurum* | Apioidae, Halictidae |  | 442 (blue), 528 (green) | Peitsch et al. (1992) |
| *Colletes fulgidus* | Apoidae, Colletidae |  | 340 (UV), 532 (green) | Peitsch et al. (1992) |
| *Cerceris rybynensis* | Apocrita, Sphecidae |  | 436 (blue), 516 (blue) | Peitsch et al. (1992) |
| *Philanthus triangulum* | Apocrita, Sphecidae | trichromatic | 344 (UV), 444 green), 524 (blue) | Peitsch et al. (1992) |
| *Dolichovespula norwegica* | Apocrita, Vespidae |  | 448 (blue), 524 (green) | Peitsch et al. (1992) |
| *Vespa cabro* | Apocrita, Vespidae | trichromatic | 336 (UV), 436 (blue), 536 (green) | Peitsch et al. (1992) |
| *Paravespula germanica* | Apocrita, Vespidae | trichromatic | 336 (UV), 432 (blue), 544 (green) | Peitsch et al. (1992) |
| *Paravespula vulgaris* | Apocrita, Vespidae | trichromatic | 336 (UV), 432 (blue), 536 (green) | Peitsch et al. (1992) |
| *Polistes gallicus* | Apocrita, Vespidae | trichromatic | 352 (UV), 452 (blue), 528 (green) | Peitsch et al. (1992) |
| *Ichneumon stramentarius* | Apocrita, Ichneumonidae |  | 524 (green) | Peitsch et al. (1992) |
| *Ichneumon sp.* | Apocrita, Ichneumonidae |  | 524 (green) | Peitsch et al. (1992) |
| *Urocerus gigas* | Symphyta, Siricidae |  | 542 (green) | Peitsch et al. (1992) |
| *Tenthredo sp.* | Symphyta, Tenthredinidae |  | 516 (green) | Peitsch et al. (1992) |
| *Tenthredo campestris* | Symphyta, Tenthredinidae | tetrachromatic | 328 (UV), 464 (blue), 540 (green), 596 (red) | Peitsch et al. (1992) |
| *Tenthredo scrophulariae* | Symphyta, Tenthredinidae |  | 532 (green), 592 (red) | Peitsch et al. (1992) |
| *Xiphydria camelus* | Symphyta, Xiphydriidae |  | 556 (green), 604 (red) | Peitsch et al. (1992) |
|  |  |  |  |  |
| **Lepidoptera** |  |  |  |  |
| *Graphium sarpedon* | Lepidoptera, Papilionidae | 15-chromatic organized in six classes | UV, V, blue, blue-green, green, red | Chen et al. (2016) |
| *Troides aeacus* | Lepidoptera, Papilionidae | nonachromic | 360 (UV), 390 (V), 440 (blue), 510 (blue-green), 540 (single-peaked green), 550 (dual-peaked green), 580 (orange), 610 (red), 630 (deep red) | Chen et al. (2006) |
| *Pieris rapae* | Lepidoptera, Pieridae | trichromatic | 360 (UV), 425 (violet), 453 (blue) | Arikawa et al. (2005) |
| *Papilio xuthus* | Lepidoptera, Papilionidae | pentachromatic | 360 (UV), 400 (violet), 460 (blue), 520 (green), 610 (red) | Arikawa et al. (1987) |
| *Papilio aegeus* | Lepidoptera, Papilionidae | tetrachromatic | 390, 450, 540, 610 | Matič (1983) |

**Table S5:** Comparison of selection differentials *S* and phenotypic selection gradients $\beta$_C_ from published studies of pollinator-mediated selection on flower color. *I.* = *Ipomopsis*.

| **Species** | **Pollinator** | **Trait** | $\boldsymbol{\beta}$**_C_** | ***S*** | **Reference** |
| --- | --- | --- | --- | --- | --- |
| **Natural populations** | |  |  |  |  |
| *I. tenuituba, I. aggregata* | Hummingbird, hawkmoth | Optical density | 0.01 ± 0.12 | -0.00 ± 0.09 | Campbell et al. (1997) |
| *I. tenuituba, I. aggregata* | Hawkmoth | Optical density | 0.08 ± 0.15 | 0.05 ± 0.14 |  |
| *Mimulus luteus* | Insects, hummingbirds | Guide shape CVA1 | 0.011 ± 0.06 | -0.040 | Medel et al. (2003) |
| *Mimulus luteus* | Insects, hummingbirds | Guide shape CVA2 | -0.007 ± 0.06 | 0.002 |  |
| *Gentiana lutea* | Bumblebees | Corolla color | **0.238 ± 0.08** | **0.281 ± 0.11** | Veiga et al. (2015) |
| *Silene littorea* | Bees, butterflies, Hadena sancta | Anthocyanin conc. | -0.11 ± 0.1 | 0.15 | Rodriguez-Castaneda et al. (2020) |
| *Silene littorea* | bees, butterflies, Hadena sancta | Corolla color | -0.1 ± 0.17 | **-0.33** |  |
| **Experimental populations** | |  |  |  |  |
| *I. tenuituba, I. aggregata* | Hummingbird, hawkmoth | Optical density | **0.18 ± 0.06** | **0.19** | Campbell et al. (1997) |

**Reference list of literature to Table S1**

Arnold, S.E.J., Savolainen, V., and Chittka, L. (2009). Flower colours along an alpine altitude gradient, seen through the eyes of fly and bee pollinators. *Arthropod-Plant Interactions* 3**,** 27-43.

Bergamo, P.J., Telles, F.J., Arnold, S.E.J., and De Brito, V.L.G. (2018). Flower colour within communities shifts from overdispersed to clustered along an alpine altitudinal gradient. *Oecologia* 188(1)**,** 223-235.

Binkenstein, J., Stang, M., Renoult, J.P., and Schaefer, H.M. (2017). Weak correlation of flower color and nectar-tube depth in temperate grasslands. *Journal of Plant Ecology* 10(2)**,** 397-405.

Campbell, D.R., Bischoff, M., Lord, J.M., and Robertson, A.W. (2012). Where have all the blue flowers gone: pollinator responses and selection on flower colour in New Zealand *Wahlenbergia albomarginata*. *Journal of Evolutionary Biology* 25**,** 352-364.

Caruso, C.M., Scott, S.L., Wray, J.C., and Walsh, C.A. (2010). Pollinators, herbivores, and the maintenance of flower color variation: a case study with *Lobelia siphilitica*. *International Journal of Plant Sciences* 171(9)**,** 1020-1028. doi: 10.1086/656511.

De Jager, M., Dreyer, L.L., and Ellis, A.G. (2011). Do pollinators influence the assembly of flower colours within plant communities? *Oecologia* 166**,** 543-553.

Dormont, L., Joffard, N., and Schatz, B. (2019). Intraspecific variation in floral color and odor in orchids. *International Journal of Plant Science* 180(9)**,** 1036-1058.

Ellis, A.G., and Johnson, S.D. (2009). The evolution of floral variation without pollinator shifts in *Gorteria diffusa* (Asteraceae). *American Journal of Botany* 96(4)**,** 793-801.

Gumbert, A., Kunze, J., and Chittka, L. (1999). Floral colour diversity in plant communities, bee colour space and a null model. *Proceedings of the Royal Society B* 266**,** 1711-1716.

Irwin, R.E., and Strauss, S.Y. (2005). Flower color microevolution in wild radish: Evolutionary response to pollinator-mediated selection. *The* *American Naturalist* 165(2)**,** 225-237. doi: 10.1086/426714.

Jersáková, J., Spaethe, J., Streinzer, M., Neumayer, J., Paulus, H., Dötterl, S., and Johnson, S.D. (2016). Does *Traunsteinera globose* (the globe orchid) dupe its pollinators through generalized food deception or mimicry? *Botanical Journal of the Linnean Society* 180(2)**,** 269-294. doi: 10.1111/boj.12364.

Joffard, N., Le Roncé, I., Langlois, A., Renoult, J., Buatois, B., Dormont, L., and Schatz, B. (2020). Floral trait differentiation in *Anacamptis coriophora*: Phenotypic selection on scents, but not on colour. *Journal of Evolutionary Biology* 33(8)**,** 1028-1038. doi: 10.1111/jeb.13657.

Kantsa, A., Raguso, R.A., Dyer, A.G., Sgardelis, S.P., Olesen, J.M., and Petanidou, T. (2017). Community-wide integration of floral colour and scent in a Meditarranean scrubland. *Nature Ecology & Evolution* 1**,** 1502-1510.

Kemp, J.E., Bergh, N.G., Soares, M., and Ellis, A.G. (2019). Dominant pollinators drive non-random community assembly and shared flower colour patterns in daisy communities. *Annals of Botany* 123**,** 277-288.

Koski, M.H., and Ashman, T-L. (2013). Quantitative variation, heritability, and trait correlations for ultraviolet floral traits in Argentina anserina (Rosaceae): Implications for floral evolution. *International Journal of Plant Science* 174(8)**,** 1109-1120.

Koski, M.H., and Ashman, T.-L. (2016). Macroevolutionary patterns of ultraviolet floral pigmentation explained by geography and associated bioclimatic factors. *New Phytologist* 211(2)**,** 708-718.

Lavi, R., and Sapir, Y. (2015). Are pollinators the agents of selection for extreme large size and dark color in *Oncocyclus* irises? *New Phytologist* 205**,** 369-377. doi: 10.1111/nph.12982.

Makino, T.T., and Yokoyama, J. (2015). Nonrandom composition of flower colors in a plant community: Mutually different co-flowering natives and disturbance by aliens. *PLoS ONE* 10(12)**,** e0143443. doi: 10.1371/journal.pone.0143443.

Meléndez-Ackerman, E.J., Campbell, D.R., and Waser, N.M. (1997). Hummingbird behavior and mechanisms of selection on flower colour in *Ipomopsis*. *Ecology* 78(8)**,** 2532-2541.

Menzel, R., and Shmida, A. (1993). The ecology of flower colours and the natural colour vision of insect pollinators: The Israeli flora a case study. *Biological Reviews* 68**,** 81-120.

Newman, E., Manning, J., and Anderson, B. (2014). Matching floral and pollinator traits through guild convergence and pollinator ecotype formation. *Annals of Botany* 113(2)**,** 373-384. doi: 10.1093/aob/mct203.

Ohashi, K., Makino, T., and Arikawa, K. (2015). Floral colour change in the eyes of pollinators: testing possible constraints and correlated evolution. *Functional Ecology* 29**,** 1144-1155. doi: 10.1111/1365-2435.12420.

Peach, K., Liu, J., Klitgaard, K.N., and Mazer, S.J. (2020). Sex-specific floral attraction in a sequentially hermaphroditic species. *Ecology and Evolution* 10**,** 1856-1875. doi: 10.1002/ece3.5987.

Schemske, D.W., and Bradshaw, H.D. (1999). Pollinator preference and the evolution of floral traits in monkeyflowers (*Mimulus*). *Proceedings of the National Academy of Sciences* 96(21)**,** 11910-11915. doi: 10.1073/pnas.96.21.11910.

Sletvold, N., Trunschke, J., Smit, M., Verbeek, J., and Ågren, J. (2016). Strong pollinator-mediated selection for increased flower brightness and contrast in a deceptive orchid. *Evolution* 70(3)**,** 716-724. doi: 10.1111/evo.12881.

Sobral, M., Veiga, T., Domínguez, P., Guitián, J.A., Guitián, P., and Guitián, J.M. (2015). Selective pressures explain differences in flower color among *Gentiana lutea* populations. *PLoS ONE* 10(7). doi: 10.1371/journal.pone.0132522.

Souto-Vilarósa, D., Vuletaa, A., Manitašević Jovanovič, S., Budečević, S., Wang, H., Sapir, Y., et al. (2017). Are pollinators the agents of selection on flower colour and size in irises? *Oikos* 127(6)**,** 834-846.

Suzuki, M.F., and Ohashi, K. (2014). How does a floral colour-changing species differ from its non-colour-changing congener? – a comparison of trait combinations and their effects on pollination. *Functional Ecology* 28**,** 549-560.

Teppabut, Y., Ovama, K., Kondo, T., and Yoshida, K. (2018). Change of petals’ color and chemical components in *Oenothera* flowers during senescence. *Molecules* 23**,** 1698. doi: 10.3390/molecules23071698.

Van der Kooi, C.J., Pen, I., Staal, M., Stavenga, D.G., and Elzenga, J.T.M. (2016). Competition for pollinators and intra-communal spectral dissimilarity of flowers. *Plant Biology* 18**,** 56-62. doi: 10.1111/plb.12328.

Veiga, T., Guitián, J., Guitián, P., Guitián, J., Munilla, I., and Sobral, M. (2015). Flower color variation in the montane plant *Gentiana lutea* L. (Gentianaceae) is unrelated to abiotic factors. *Plant Ecology & Diversity*. doi: 10.1080/17550874.2015.1074626.

Weiss, M.R. (1995). Floral color change: A widespread functional convergence. *American Journal of Botany* 82(2)**,** 167-185.

Zhang, C., Vereecken, N.J., Wang, L. Tian, B., Dafni, A., Yang, Y., and Duan, Y. (2017). Are nectar guide colour changes a reliable signal to pollinators that enhances reproductive success? *Plant Ecology & Diversity* 10**,** 89-96. doi: 10.1080/17550874.20171350763.

**Reference list of literature to Table S2**

Aldridge, G., and Campbell, D.R. (2007). Variation in pollinator preference between two *Ipomopsis* contact sites that differ in hybridization rate. *Evolution* 61(1)**,** 99-110.

Campbell, D.R., Waser, N.M., and Meléndez-Ackerman, E.J. (1997). Analyzing pollinator-mediated selection in a plant hybrid zone: Hummingbird visitation patterns on three spatial scales. *American Naturalist* 149(2), 295-315. doi: 10.1086/285991.

De Jager, M.L., Dreyer, L.L., and Ellis, A.G. (2011). Do pollinators influence the assembly of flower colours within plant communities? *Oecologia* 166**,** 543-553. doi:10.1007/s00442-010-1879-7.

Faegri, K., and van der Pijl, L. (1979). The principles of pollination ecology. *Oxford; New York: Pergamon Press.*

Fenster, C.B., Armbruster, W.S., Wilson, P., Dudash, M.R., and Thomson, J.D. (2004). Pollination syndromes and floral specialization. *Annual Review of Ecology Evolution and Systematics* 35**,** 375-403. doi: 10.1146/annurev.ecolsys.34.011802.132347.

Hirota, S.K., Nitta, K., Kim, Y., Kato, A., Kawakubo, N., Yasumoto, A.A., and Yahara, T. (2012). Relative role of flower color and scent on pollinator attraction: Experimental tests using F1 and F2 hybrids od daylily and nightlily. *PLoS ONE* 7(6)**,** e39010. doi: 10.1371/journal.pone.0039010.

Hopkins, R., and Rausher, M.D. (2012). Pollinator-mediated selection on flower color allele drives reinforcement. *Science* 335**,** 1090-1092.

Johnson, S.D., and Midgley, J.J. (1997). Fly pollination of *Gorteria diffusa* (Asteraceae), and a possible mimetic function for dark spots on the capitulum. *American Journal of Botany* 84(4)**,** 429-436.

Newman, E., Anderson, B., and Johnson, S.D. (2012). Flower colour adaptation in a mimetic orchid. *Proceedings of the Royal Society B* 279(1737)**,** 2309-2313. doi: 10.1098/rspb.2011.2375.

Newman, E., Manning, J., and Anderson, B. (2014). Matching floral and pollinator traits through guild convergence and pollinator ecotype formation. *Annals of Botany* 113(2)**,** 373-384. doi: 10.1093/aob/mct203.

Schemske, D.W., and Bradshaw, H.D. (1999). Pollinator preference and the evolution of floral traits in monkeyflowers (*Mimulus*). *Proceedings of the National Academy of Sciences* 96(21)**,** 11910-11915. doi: 10.1073/pnas.96.21.11910.

Streisfeld, M.A., and Kohn, J.R. (2007). Environment and pollinator-mediated selection on parapatric floral races of *Mimulus aurantiacus*. *Journal of Evolutionary Biology* 20(1)**,** 122-132. doi: 10.1111/j.1420-9101.2006.01216.x.

Tastard, E., Ferdy, J.-B., Burrus, M., Thébaud, C., and Andalo, C. (2012). Patterns of floral colour neighbourhood and their effects on female reproductive success in an *Antirrhinum* hybrid zone. *Journal of Evolutionary Biology* 25**,** 388-399.

Whithead, M.R., Gaskett, A.C., Johnson, S.D. (2019). Floral community predicts pollinators’ color preference: implications for Batesian floral mimicry. *Behavioral Ecology* 30(1), 213-222. doi: 10.1093/beheco/ary138.

**Reference list of literature to Table S3**

Chittka, L. (1992). The colour hexagon: a chromaticity diagram based on photoreceptor excittions as a generalized representation of colour opponency. Journal of Comparative Physiology A 170, 533-543. doi: 10.1007/BF00199331.

Geyer, C.J. (2007). Aster models for life history analysis. Biometrika 94, 415-426.

Grace, J.B. (2006). Structural equation modelling in natural systems. Cambridge University Press, Cambridge.

Henshaw, J.M., and Zemel, Y. (2017). A unified measure of linear and nonlinear selection on quantitative traits. Methods in Ecology and Evolution 8, 604-614. doi: 10.1111/2041-210X.12685.

Sandring, S., and Ågren, J. (2009). Pollinator-mediated selection on floral display and flowering time in the perennial herb Arabidopsis lyrata. Evolution 63(5), 1292-1300.

Shaw, R.G. (2008). Unifying life history analysis for inference of fitness and population growth. American Naturalist 172, E35-47.

**Reference list of literature to Table S4**

Arikawa, K. (1987). Pentachromatic visual system in a butterfly. *Naturwissenschaften* 74**,** 297-298.

Arikawa, K., Wakakuwa, M., Qiu, X., Kurasawa, M. and Stavenga, D.G. (2005). Sexual dimorphism of short-wavelength photoreceptors in the Small White Butterfly, *Pieris rapae crucivora*. *Journal of Neuroscience* 25(25)**,** 5935-5942.

Chen, P.-J., Awata, H., Matsushita, A., Yang, E.-C., and Arikawa, K. (2016). Extreme spectral richness in the eye of the common bluebottle butterfly, *Graphium sarpedon*. *Frontiers in Ecology and Evolution* 4**,** 18. doi: 10.3389/fevo.2016.00018.

Chen, P.-J., Arikawa, K., and Yang, E.-C. (2013). Diversity in the photoreceptors and spectral opponency in the compound eye of the Golden Birdwing, *Troides aeacus formosanus*. *PLoS ONE* 8**,** e62240. doi: 10.1371/journal.pone.0062240.

Frantsevich, L., Govardovski, V., Gribakin, F., Nikolajev, G., Pichka, V., Polansky, A., Shevchenko, V., and Zolotov, V. (1977). Astroorientation in *Lethrus* (Coleoptera, Scarabaeidae). *Journal of Comparative Physiology* 121**,** 253-271.

Hardie, R., Vogt, K., and Rudolph, A. (1989). The compound eye of the tsetse fly (*Glossina morsitans morsitans* and *Glossina palpalis palpalis*). *Journal of Insect Physiology* 35(5)**,** 423-431.

Hertel, H., and Ventura, D.F. (1985). Spectral sensitivity of photoreceptors in the compound eye of stingless tropical bees. *Journal of Insect Physiology* 21(12)**,** 931-935.

Horridge, G.A., Mimura, K., and Tsukahara, Y. (1975). Fly photoreceptors – II. Spectral and polarized light sensitivity in the drone fly *Eristalis*. *Proceedings of the Royal Society B* 190(1099)**,** 225-237.

Martínez-Harms, J., Vorobyev, M., Schorn, J., Shmida, A., Keasar, T., Homberg, U., et al. (2012). Evidence of red sensitive photoreceptors in *Pygopleurus israelitus* (Glaphyridae: Coleoptera) and its implications for beetle pollination in the southeast Mediterranean. *Journal of Comparative Physiology A* 198(6)**,** 451-463.

Matič, T. (1983). Electrical inhibation in the retina of the butterfly *Papilio*. I Four spectral types of photoreceptors. *Journal of Comparative Physiology* A 152**,** 169-182.

Menzel, R., Ventura, D.F., Hertel, H., de Souza, J.M., and Greggers, U. (1986). Spectral sensitivity of photoreceptors in insect compound eyes: Comparison of species and methods. *Journal of Comparative Physiology* 158**,** 165-177.

Peitsch, D., Fietz, A., Hertel, H., de Souza, J., Ventura, D.F., and Menzel, R. (1992). The spectral input systems of hymenopteran insects and their receptor-based colour vision. *Journal of Comparative Physiology A* 170**,** 23-40.

von Helversen, O. (1972). Zur spektralen Unterschiedsempfindlichkeit der Honigbiene. *Journal of Comparative Physiology A* 80**,** 139-472.
